# Complete stereochemical control to unlock monosign circularly polarised luminescence with superior circularly polarised brightness for chameleon security inks

**DOI:** 10.1039/d5sc05303j

**Published:** 2026-01-12

**Authors:** Artemijs Krimovs, Dominic J. Black, Aileen Congreve, Robert Pal

**Affiliations:** a Department of Chemistry, Durham University South Road Durham DH1 3LE UK robert.pal@durham.ac.uk

## Abstract

A novel arylalkynylpyridine-sensitised nine coordinate quasi-*C*_3_ symmetric all carboxylate donor europium(iii) complex (EuL) possessing exceptionally high circularly polarised brightness in both Δ*J* = 1 and Δ*J* = 2 transitions was prepared and tested in spin-coated solid-state PMMA thin films. The authentication of the circularly polarised luminescence (CPL) layer was successfully performed using CPL photography (CPLP) and enantioselective differential chiral contrast (EDCC) imaging for both transitions simultaneously using appropriate band pass filters. The effect of reflective properties of different thin film substrate materials on the recorded chiral contrast was quantified using the newly introduced CPLP dissymmetry factor (*g*_CPLP_) which compared to the average dissymmetry factor values obtained using a photo elastic modulator (PEM) based CPL spectrometer. Circularly polarised brightness (CPB) of Δ*J* = 2 (590 mol^−1^ dm^3^ cm^−1^ at 607 nm) was the highest ever reported and that of Δ*J* = 1 (307 mol^−1^ dm^3^ cm^−1^ at 596 nm) was third best across other CPL-active materials with reported CPB. This makes EuL the best candidate for next-generation CPL-active multi-tier ‘chameleon security inks’.

## Abstract 2

A novel arylalkynylpyridine-sensitised quasi-*C*_3_ symmetric all carboxylate donor Eu(iii)-9N3 complex (EuL) possessing exceptionally high CPL brightness in both Δ*J* = 1 & 2 transitions was prepared and studied in spin-coated solid-state PMMA films.

## Graphical abstract

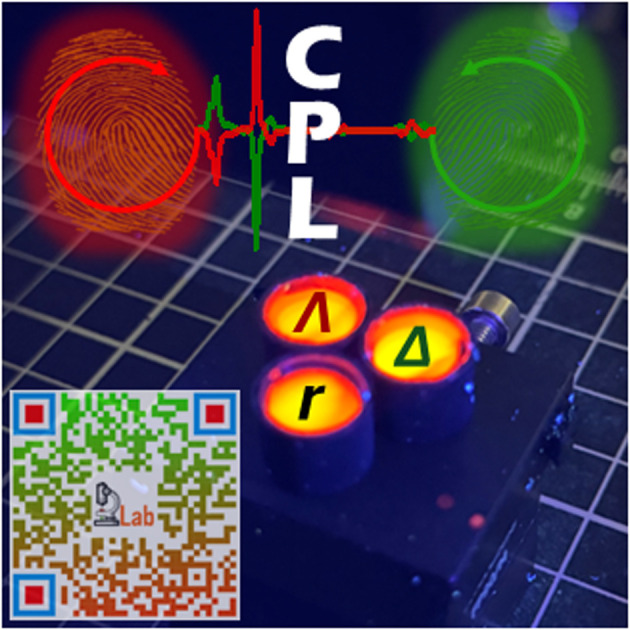

## Introduction

Light emitting materials that possess circularly polarised luminescence (CPL) have been increasingly developed for security applications.^1–4^ CPL-based security layer could improve the existing anti-counterfeiting technologies for the use in passports, driving-licenses and banknotes.^5–16^ The strength of CPL is characterised by luminescence dissymmetry factor (*g*_lum_):1
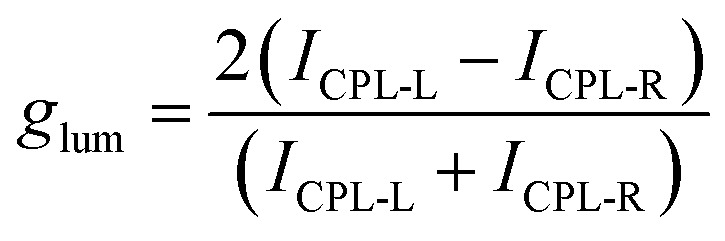
where *I*_L_ and *I*_R_ are intensities of left-handed and right-handed components of CPL. The values of *g*_lum_ range between *g*_lum_ = 2 (100% left-handed polarisation) and *g*_lum_ = −2 (100% right-handed polarisation), where *g*_lum_ = 0 indicates no net circular polarisation (CP). Practically useful CPL-active dyes must not only have high *g*_lum_ but also luminescence brightness (*B*) which is a product of molar absorption coefficient (*ε*) and photoluminescence quantum yield (*φ*). The product of the two is known as CPL brightness (CPB):^17^2
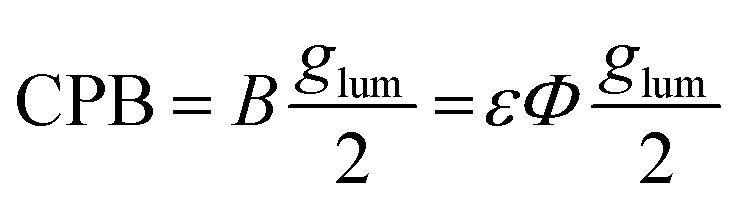


CPB can also be used as a reference parameter to compare the performance of different CPL-active materials, where the maximal observed value of the *g*_lum_ is used. CPB can also be calculated for individual transition (CPB_*i*_) using branching factor (*β*_*i*_) which is the ratio between the intensities of the emission band of interest (*I*_*i*_) and total emission:^17^

3 CPB_*i*_ = *β*_*i*_ × CPB

It is particularly useful for applications where the wavelength of the detected light can be selected, for instance, by a band pass filter (BPF).^18^ In that case, the average *g*_lum_ for the selected wavelength region is used to calculate CPB_*i*_. Therefore, the emission manifold with single CPL sign is desired to maximise CPB_*i*_ by avoiding cancellation of CP. Therefore, materials with high CPB_*i*_ can be used in security tags suitable for rapid CPL verification.

CPL-active materials with the highest known CPB values are organometallic lanthanide complexes.^17^ Here we explore arylalkynylpyridine-sensitised nine-coordinate quasi-*C*_3_ symmetric 9N3 europium(iii) complexes.^19–27^ These already found applications in bioimaging due to their advantageous photophysical properties such as millisecond long emission lifetime, narrow fingerprint like lanthanide-centred f–f emission bands, large *pseudo*-Stokes' shift and tunability of sensitisation *via* the ligand structure optimisation of absorption maxima and targeted cell localisation.^22,23^

Eu(iii) complexes have been explored as candidates for CPL-active security inks, that can be authenticated by both enantioselective differential chiral contrast (EDCC) imaging using CPL microscopy and circularly polarised luminescence photography (CPLP) using a novel handheld CPLP camera (SI Section 2.3).^18,28^ The latter exploits a narrow 10 nm BPF for isolation of individual transitions, for example, 594 ± 5 nm BPF (BPF594/10) and 610 ± 5 nm BPF (BPF610/10) to select ^5^D_0_ → ^7^F_1_ (Δ*J* = 1) and ^5^D_0_ → ^7^F_2_ (Δ*J* = 2) respectively. Commonly, only magnetic-dipole (MD) allowed Δ*J* = 1 transition has been used for CPL-imaging due to its strong CPL activity and single CPL sign conservation. The use of Δ*J* = 1 transition is not optimal since it makes up only around 5% of total EuL emission. The use of the brightest Δ*J* = 2 (*β*_*i*_ = ∼35%) is more desirable since it would require around 7 times less material to produce the same emission intensity and therefore CPB_*i*_. On the other hand, due to its MD forbidden nature, Δ*J* = 2 usually shows low *g*_lum_. In addition to that, the environmentally hypersensitive Δ*J* = 2 manifold often shows complex variation in CPL sign that result in overall cancellation of the detected CPL signal.^29^ Both factors significantly reduce CPB_*i*_ of Δ*J* = 2, making it unsuitable for CPLP.

This leads to the main requirement for a CPL-active security ink: conservation of the CPL sign across the emission manifold to maximise the average *g*_lum_ within the selected wavelength region. Therefore, it is important to consider the symmetry at the Eu(iii) emitting centre that affects the multiplicity of the electronic states involved in a transition. For Eu(iii), the emissive ^5^D_0_ state has a multiplicity of 1 (*J* = 0) in all symmetries unlike the multiplicities of the ^7^F_1_ and ^7^F_2_ which depend on the point group. As a result, a lower symmetry at Eu(iii) increases the multiplicity of the two states which then leads to multiple CPL emission bands within Δ*J* = 1 and Δ*J* = 2 with potentially opposing helicity.

The symmetry at Eu(iii) site in the complexes of interest is attributed to the *C*_3_ point group, which suggests the multiplicity of 2 and 3 for the ^7^F_1_ and ^7^F_2_ states respectively. This multiplicity is the same across other high symmetry classes such as hexagonal and octagonal but can increase when the symmetry is lowered.

Parent Eu(iii) complex structures that were the most widely explored for their CPL activity contained phosphinate donor groups.^2,20,28,30^ The use of pentavalent phosphorus allows for additional structural modifications. For example, bulky phenyl groups on phosphorus are believed to limit complex racemisation since it requires simultaneous inversion of the chiral phosphorus centre as well as inversion of the 1,4,7-triazacyclononane (TACN) ring and rotation of the sensitising chromophores.^2^ This is important since enantiopure complexes must not racemise during short-term exposure to high temperature (up to 150 °C) during lamination. On the contrary, phosphinate donor complexes normally possess multiple low intensity CPL bands in the Δ*J* = 2 manifold which often demonstrate CPL sign fluctuation. A recent exception to this was a mixed-donor complex containing two phosphinate and one carboxylate donor groups.^4^ Although it produced a single-sign CPL Δ*J* = 2 manifold, it only contained two sensitising chromophores (out of 3 possible) reducing *ε* and therefore inherently reducing CPB. Although a direct relationship between the complex structure and its observed CPL spectrum is not fully understood, it was proposed that substitution of chiral phosphinate donors with achiral centrosymmetric bidentate carboxylate donors would reduce the magnetic dipole moment induced (total angular momentum driven). This would greatly affect the overall rotatory strength of the molecule and the variation on CPL sign within the Δ*J* = 1 and Δ*J* = 2 manifold. In contrast to the pseudo-tetrahedral geometry of the phosphinate donor, trigonal planar geometry of the carboxylate donor does not allow the oxygen atom to approach the luminescent Eu^3+^ centre as closely. This leads to a weaker crystal field experienced by Eu^3+^ which reduces the crystal field splitting of the individual ^7^F_*J*_ electronic states (especially relevant for the ‘hypersensitive’ Δ*J* = 2 transition). As a result, the transition multiplet is simplified on both the total emission spectrum and the CPL spectrum. This increases the probability of the CPL sign conservation within a transition by decreasing the total number of individual bands that can produce sequentially opposite sign of CPL.

In this work, the design of the novel EuL complex was aimed on conservation of the CPL sign within both Δ*J* = 1 and Δ*J* = 2 transition manifolds. This will make the EuL the best known candidate for the recently proposed Chameleon Security Inks (CSI) concept comprising of a blend of achiral short-lived (ns) luminescent dyes and chiral (or achiral) long-lived (ms) europium(iii) emitters.^18^ Strong dissymmetry of the Δ*J* = 2 would generate an additional CPL security layer to the existing five-tier multi-coloured, multi-spectral, opposing helicity security, combined with high spatial and temporal resolution. In addition, the ability to use the brightest Δ*J* = 2 transition for CPL based security authentication would reduce the required amount of the material and therefore the resulting cost of the security tag, bringing it another step closer towards widespread commercial application.

## Results and discussion

### Synthesis of EuL

**Fig. 1:**
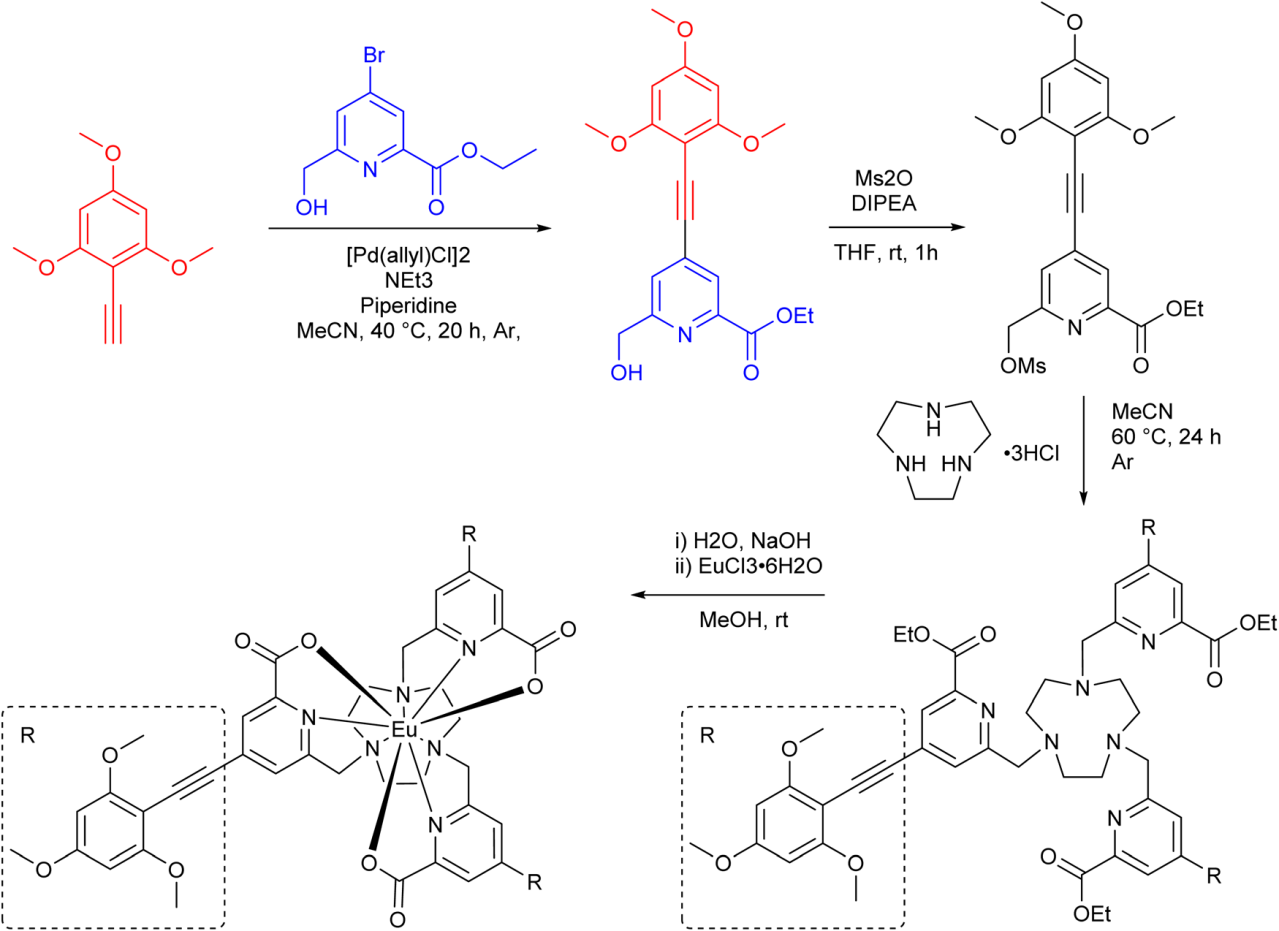
Reaction scheme for the synthesis of EuL from the arylalkynyl (red) and bromo pyridyl (blue) components of the chromophore.

The synthetic design of EuL was based on the examples of the previously reported modular approach for isostructural materials with details provided in the supplementary information (SI Section 6).^19,20,22,24,27^ The ‘top’ aryl-alkynyl (Fig. 1 – red) and the ‘bottom’ pyridyl (Fig. 1 – blue) components of the sensitising chromophore are synthesised separately (SI Fig. 5 and 6) before they are coupled in a Sonogashira reaction. This provides a functionalisation flexibility to achieve the desired photophysical properties. The chromophore is then mesylated and attached to the 1,4,7-triazacyclononane (TACN) to form a macrocycle containing three chromophores. This if followed by a base-catalysed hydrolysis and complexation to a europium(iii) chloride hexahydrate to produce EuL as a racemic mixture. The two enantiomers of EuL were then separated using chiral HPLC (SI Section 3).

### General photophysical properties

**Fig. 2:**
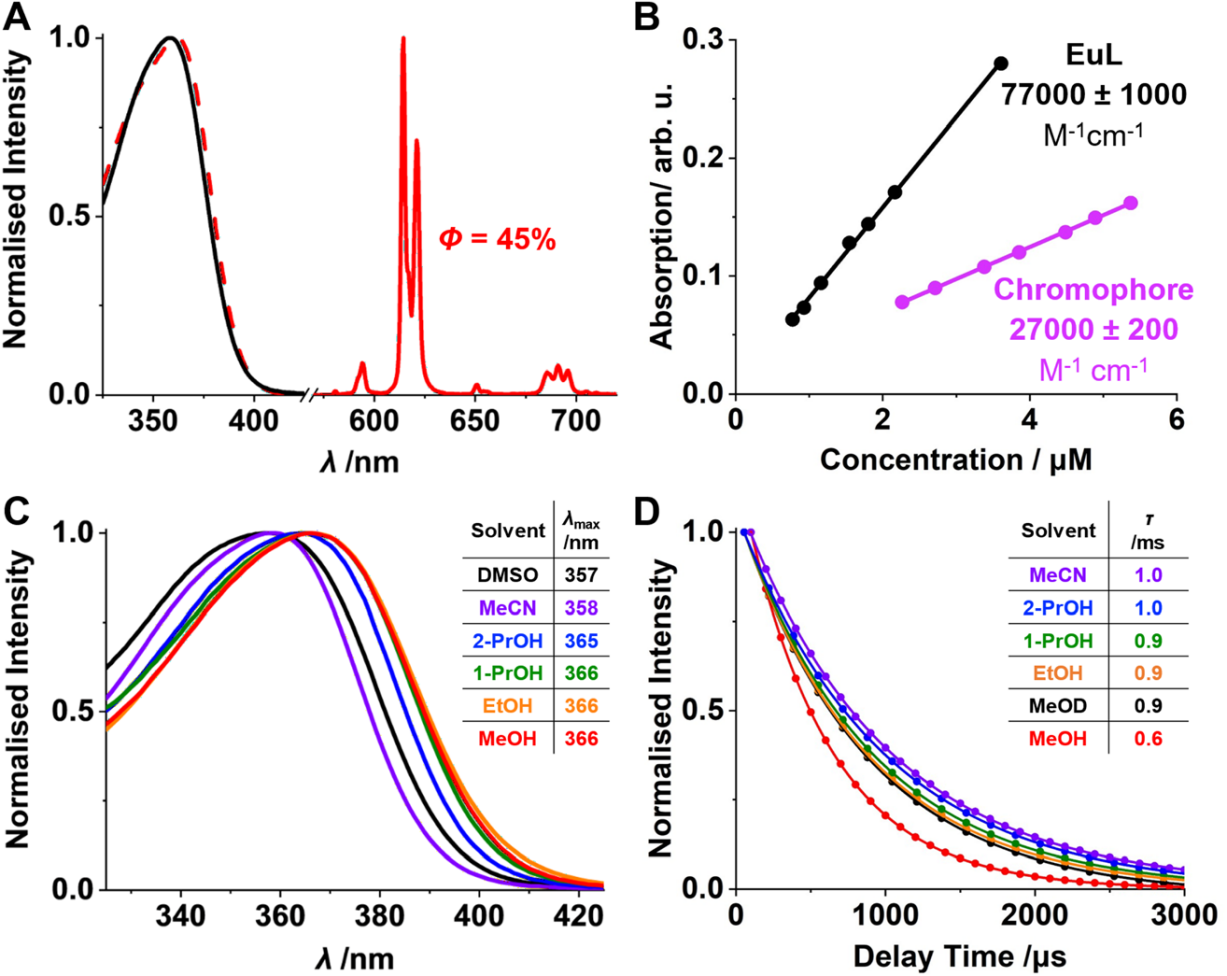
(A): Normalised absorption (black), excitation (dashed red, *λ*_em_ = 615 nm) and emission (red, *λ*_exc_ = 360 nm) spectra and *Φ* of EuL in MeCN. (B): Absorption against concentration of EuL and its chromophore in MeCN. (C): Normalised absorption spectra of EuL in solvents of increasing polarity. (D): Normalised fluorescence emission lifetime of EuL in solvents of increasing polarity [EuL] = 1 µM.

Similarly to other sensitised lanthanide complexes, a large pseudo-Stokes shift of around 257 nm was observed for EuL (Fig. 2A). The band shape match between the absorption and excitation spectra of EuL in MeCN confirmed the sensitised nature of the Eu(iii) emission. Importantly, the emission profile was independent of the solvent, which is advantageous for wide security application, where different host material can be used. The quantum yield (*Φ*) of EuL was measured as 45% in acetonitrile (MeCN) using the absolute method (SI Section 2). The molar extinction coefficient (*ε*) of EuL was determined as 77 000 ± 1000 M^−1^ cm^−1^ in MeCN (Fig. 2B). In order to confirm the accuracy, the *ε* of a single chromophore was measured as 27 000 ± 200 M^−1^ cm^−1^ in MeCN which was approximately one third of that measured for EuL containing three chromophores.

EuL is sensitised by arylalkynyl pyridyl containing chromophores that are known to possess a photoinduced internal charge transfer (ICT) excited state.^31^ This is provided by an electron rich aryl and electron poor pyridyl groups conjugated by the alkyne linker. It was previously reported that the highest occupied molecular orbital (HOMO) of such chromophore is localised on the electron-rich aryl.^21^ Therefore, substitution of the aryl with electron-donating groups results in a bathochromic shift of the absorption maximum (*λ*_max_) mediated by an increased HOMO energy.^19^ This is beneficial for the application in security inks where longer excitation wavelengths such as that of commercially available 365 nm light emitting diodes (LEDs). As a result, *λ*_max_ of EuL in MeCN was recorded at 358 nm (Fig. 2C), with still 94% of maximal absorbance at 365 nm. A bathochromic shift of *λ*_max_ with increasing solvent polarity was also observed, which was in agreement with the ICT nature of the transition.^32^

No strong correlation between the emission lifetime (*τ*) and solvent polarity was observed for the chosen group of solvents with *τ* values ranging between 1.0 and 0.9 ms at room temperature (Fig. 2D). A significantly lower *τ* of 0.6 ms was recorded in methanol (MeOH). The experiment was repeated in deuterated methanol (MeOD) to produce a higher *τ* of 0.9 ms. Since *τ* obtained in MeOD was similar to the rest of the other alcohols, emission quenching was not attributed to vibrational relaxation of the chromophore excited state *via* O–H oscillators. Instead, the difference in *τ* was attributed to hydrogen bonding ability of the solvent, where the carboxylate donor group of the chromophore can act as a hydrogen bond acceptor. This decreases the energy of the ICT excited state resulting in a higher rate of the thermally activated back energy transfer (BET) from the excited ^5^D_0_ state of Eu(iii). This increases the lifetime of the ICT excited state and therefore the probability of nonradiative relaxation processes.

### Circularly polarised luminescence spectroscopy and circularly polarised brightness

**Fig. 3:**
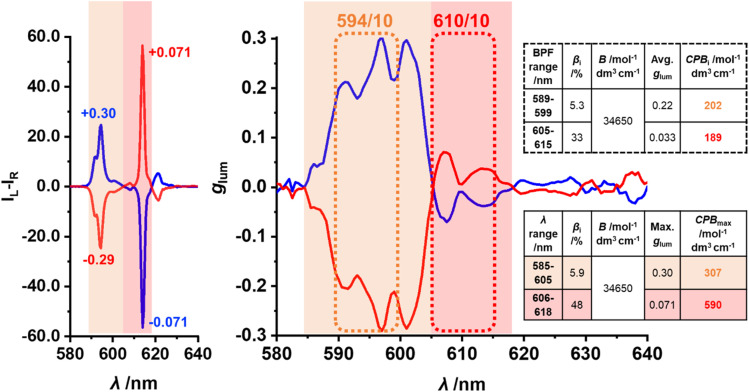
CPL spectra with maximal *g*_lum_ values for Δ*J* = 1 and Δ*J* = 2 shown (left) and the corresponding *g*_lum_ plots (right) of *Δ*-EuL (blue) and *Λ*-EuL (red) in MeCN (*λ*_ex_ = 360 nm, 5 averages) and their maximal with the *λ* ranges used for CPB calculations shown and the relevant values summarised in the inset tables.

The two enantiomers of EuL produced mirrored CPL spectra of opposite signs. The enantiomers were assigned as *Δ* and *Λ* based on the CPL sign of the Δ*J* = 1 transition, which was in accordance with the previously reported X-ray crystallographic study of isostructural materials.^33^ The racemisation study of EuL in different solvents at 60 °C determined racemisation half-life of 190 ± 20 hours (SI Section 3, SI Fig. 3), while no racemisation was observed at room temperature. This suggests that EuL would be suitable for security applications with long-term conservation of CPL dissymmetry at ambient conditions and short-term stability towards racemisation during document lamination (150 °C, 1 s cm^−1^).^18^ In addition, CPL profile was independent on solvent (SI Fig. 7) suggesting suitability for a wide-spread application in different host materials with conservation of CPL properties. In contrast to the previously reported CPL spectra of various phosphinate donor complexes,^4,18,20,29^ the Δ*J* = 2 band was highly CPL active with significant sign retention (Fig. 3). This resulted in the peak CPL intensity of the Δ*J* = 2 (at 615 nm) around 2.5 times higher than that of Δ*J* = 1 (at 594 nm). The only example with such strongly CPL-active monosign Δ*J* = 2 band is Eu:BPEPC, a commonly known CPL-standard.^34^ The whole Δ*J* = 1 manifold (588–605 nm) of EuL was single-sign, producing the average *g*_lum_ of 0.21 and the highest *g*_lum_ values of +0.30 (at 597 nm) and +0.29 (at 601 nm) for *Δ*-EuL and −0.29 (at 597 nm) and −0.29 (at 601 nm) for *Λ*-EuL (Fig. 3). Such outstanding *g*_lum_ values of EuL are higher than those of most other reported Eu(iii) complexes Although there are few examples that report Eu(iii) complexes with higher than 0.30 *g*_lum_ values for the Δ*J* = 1 transition, they often have lower values of *Φ* and *ε*, resulting in lower CPB_*i*_ which limits their security application.^17,35^ For example, one material demonstrated the *g*_lum_ values of ±0.33 (at 600 nm) but a relatively low *Φ* of 11%.^36^ Another example reported similar *g*_*l*um_ values of +0.298 and −0.294 but much lower *ε* (23 000 mol^−1^ dm3 cm^−1^) and *Φ* (11%).^37^ The material (Cs[Eu(+)-(hfbc)_4_]) with the highest ever reported *g*_lum_ of 1.38 (at 595 nm) also has significantly lower *ε* (35 000 mol^−1^ dm^3^ cm^−1^) and *Φ* (3%).^38^ This highlights the superiority of CPB_*i*_ over glum to assess the performance of the CPL emitters for practical applications.

The average *g*_lum_ of the single-sign region within the Δ*J* = 2 manifold (606–618 nm) was 0.031 with the highest values of −0.071 (at 607.5 nm) for *Δ*-EuL and +0.071 (at 607 nm) for *Λ*-EuL. This puts EuL the third best in terms of previously reported *g*_lum_ for Δ*J* = 2 after previously mentioned Cs[Eu(+)-(hfbc)_4_] producing *g*_lum_ of 0.25 (at 614 nm) and another material with 0.11 at (616 nm).^34,38^ The latter also has lower values of *ε* (55 000 mol^−1^ dm^3^ cm^−1^) and *Φ* (11%) which reduces the CPB_*i*_.

In order to assess the applicability of EuL for the use in security tags authenticated by CPLP, CPB_*i*_ for the single-sign regions of Δ*J* = 1 and Δ*J* = 2 CPL bands were estimated. To account for the variation of the CPL intensity and *g*_lum_ within the selected (by BPFs) wavelength regions, the average *g*_lum_ value for each region was used to calculate CPB_*i*_ (Fig. 3, top table). As a result, the calculated CPB_*i*_ for Δ*J* = 1 and Δ*J* = 2 transitions were similar (202 and 189 mol^−1^ dm^3^ cm^−1^), suggesting both transitions can be used for CPLP. Since previously reported CPB values for other materials were calculated using the maximal *g*_lum_ for each transition, they were also calculated as CPB_max_ (Fig. 3, bottom table) for comparative study. As a result, the maximal CPB_*i*_ for Δ*J* = 1 and Δ*J* = 2 were calculated as 307 (at 596 nm) and 590 (at 607 nm) mol^−1^ dm^3^ cm^−1^ respectively. This puts CPB_*i*_ of EuL above the average for both Δ*J* = 1 and Δ*J* = 2 (286.6 and 69.4 mol^−1^ dm^3^ cm^−1^ respectively) in previously reported CPL-active Eu^3+^ complexes.^17^ EuL is the third best in terms of the CPB_*i*_ for Δ*J* = 1 after the recently reported tetrahedral Eu_4_L_4_(L′)_4_ cages with CPB_*i*_ of 3240 and 1122 mol^−1^ dm^3^ cm^−1^.^39^ On the other hand, CPB_*i*_ for the Δ*J* = 2 transition in these materials was not reported. The unprecedented CPB_*i*_ of 590 mol^−1^ dm^3^ cm^−1^ (at 607 nm) produced by the Δ*J* = 2 of EuL is the highest ever reported to the knowledge of the author with the second best producing almost three times lower value of 213 mol^−1^ dm^3^ cm^−1^.^34^

### Circularly polarised luminescence photography

The proposed security tags containing enantiopure EuL would be authenticated with the recently developed CPL photography (CPLP) camera (SI Section 2.3).^18^ Its working principle is based on precise alignment of a quarter waveplate (QWP) with 4 different orientations (0°, 45°, 90° and −45°) linear polarisers covering an array of photodiodes. QWP converts CPL into a linearly polarised (LP) light with polarisation plane at 45° (right-hand CPL (RCPL)) and −45° (left-hand CPL (LCPL)) with respect to the fast axis. This LP light then passes through the polarisers before sensitising the underlying photodiodes. This generates sensitivity of the camera towards the CPL sign where 4 simultaneously taken images contain different information on chirality of the light emitted by the probe. For example, if the QWP is aligned with the 45° polarisers, then 0° and 90° channels will be more sensitive towards LCPL (L image) and RCPL (R image) respectively while 45° and −45° will each contain half of the total emitted light intensity. Since chiral probes still emit both LCPL and RCPL, image subtraction is required to generate a ‘true’ CPL image, which was previously reported as enantioselective differential chiral contrast (EDCC).^28^ For example, to obtain a CPL image of a LCPL-active probe, the intensity of the R image can be subtracted from the intensity of the L image (L − R). Since the Fiji (version 1.53q) software^40^ doesn't allow for negative intensity, the R − L image of such probe will be zero rather than negative (practically near zero due to solarisation dependent pixel displacement and directional light guided reflection). Therefore, for RCPL-active probes the R − L image will be relevant. The total intensity can be calculated by L + R and used to obtain the CPLP specific dissymmetry factor (*g*_CPLP_):4
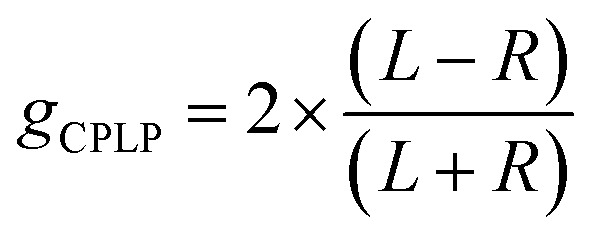


Importantly, when R − L image was used in calculation, the *g*_CPLP_ value must be multiplied by −1 to account for the negative CPL sign of RCPL:5
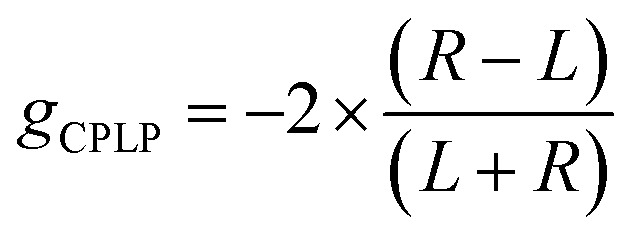


The choice of the correct formula is based on relative intensity of the L − R and R − L images, where the brightest image is used.

In theory, *g*_CPLP_ must correlate with *g*_lum_ due to a similar light polarisation information acquisition principle between the CPLP camera and photo elastic modulator (PEM) based CPL spectrometer. In the CPLP camera blueprint the QWP is fixed and orientations of the linear polariser clusters are used to distinguish the L- and R-CPL, whilst the conventional CPL spectrometer exploits a PEM that serves as a QWP with a variable angular orientation in combination with a fixed linear polariser.

**Fig. 4:**
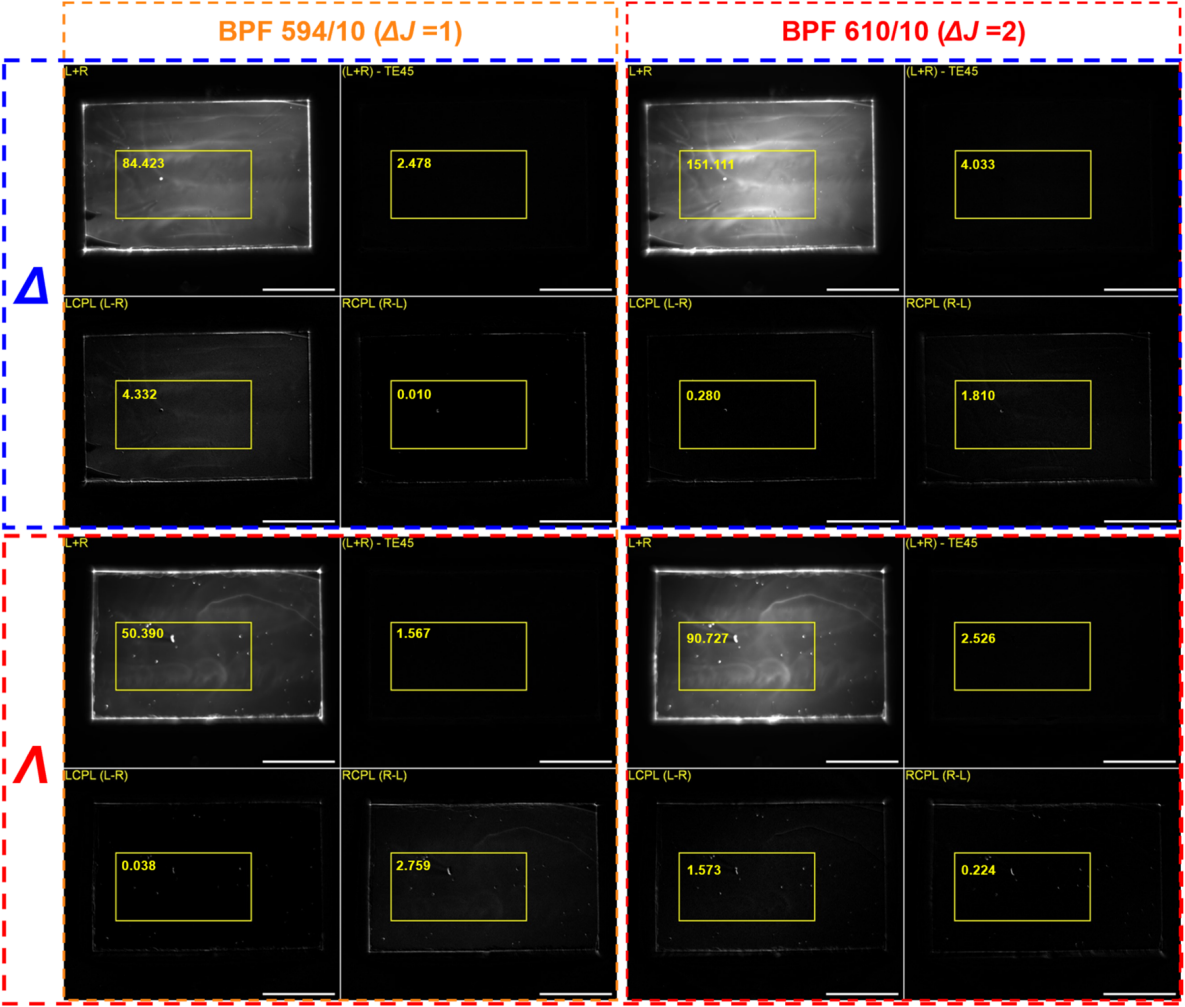
CPLP calculated images (L + R, (L + R)-TE45*, L − R and R − L) of the spin coated PMMA films under 365 nm excitation containing Δ (top two rows) and Λ (bottom two rows) enantiomers of EuL_1_ using BPF594/10 (two left columns) and BPF610/10 (two right columns) with regions of interest for *g*_CPLP_ calculations and their average intensities shown in yellow. Scale bar = 5 mm. R − L and L − R images are 6 times contrast enhanced for display.*TE45 image is a sum of the two images generated from 45° and −45° channels that have no CPL sign preference (QWP aligned with 45° polarisers) and record half of the total intensity each; subtraction of TE45 from (L + R) should theoretically produce an image with zero intensity, which is practically nonzero due to experimental error.

As a result, not only the observed CPL sign but also *g*_CPLP_ numerical values could be used to authenticate a CPL-active security ink. To test the CPL security performance of EuL, polymethyl methacrylate (PMMA) thin films (200 nm) containing the two enantiomers (labelled as *Δ* and *Λ*) were spin coated on glass (SI Section 4) and subjected to CPLP followed by EDCC (Fig. 4). Due to the similarity between the obtained CPB_*i*_ values of 202 and 189 mol^−1^ dm^3^ cm^−1^ for the wavelength regions of BPF594/10 and BPF610/10, both Δ*J* = 1 and Δ*J* = 2 transitions were imaged. The L + R, L − R and R − L calculated images were then used for *g*_CPLP_ calculations. In contrast to measuring the intensity of the whole image, a specific region of interest was chosen to avoid the error associated with rough edges of the glass substrate which is a source of undesired reflections of the emitted light.

As expected, each enantiomer produced intensity in either L − R or R − L depending on the BPF used. This correlated with the CPL sign of each enantiomer within the selected wavelength regions of the Δ*J* = 1 and Δ*J* = 2 manifolds. For example, the CPL spectrum of *Δ*-EuL has a positive sign in 589–599 nm region (BPF594/10, Δ*J* = 1) and a negative sign in 605–615 nm region (BPF610/10, Δ*J* = 2), which correlated with L − R and R − L images when using BPF594/10 and BPF = 610/10 respectively. The *g*_CPLP_ values were then calculated from the L − R and R − L images as +0.10 and −0.0024 respectively. When compared to the average *g*_lum_ values recorded using a PEM-CPL spectrometer for the equivalent wavelength regions (+0.21 for 589–599 nm and −0.033 for 605–615 nm and), it was evident that the magnitude of dissymmetry factor produced by the *Δ*-EuL containing film in Δ*J* = 1 and Δ*J* = 2 decreased by ∼50% and ∼30%. Similar results were obtained for the film containing *Λ*-EuL, where the *g*_CPLP_ value of −0.11 decreased by ∼50% compared to *g*_lum_ of −0.22 for Δ*J* = 1; however, remained essentially the same for the Δ*J* = 2 where *g*_CPLP_ = +0.035 and *g*_lum_ = +0.033.

To confirm that the reduction in *g*_CPLP_ is not a product of a random error, the calculation was repeated for multiple films that were sequentially spin coated using the same method as for the original film (SI Fig. 10 and 11). This could also determine whether the structural imperfections of the spin-coated films significantly impact the *g*_CPLP_.

As a result, all 4 films consistently produced nearly identical *g*_CPLP_ values when using the same BPF, resulting in 50% and 30% *g*_CPLP_ reduction for the Δ*J* = 1 (BPF594/10) and Δ*J* = 2 (BPF610/10) respectively compared that of the *Δ*-EuL solution (Table 1). It was hypothesised that such reduction in *g*_CPLP_ resulted from reflection of CPL from the glass substrate that led to the sign inversion. The stronger effect observed in the 589–599 nm region could be attributed to shorter wavelength light being more susceptible to scattering compared to that of longer wavelength.

**Table 1:** Comparison of the *g*_CPLP_ of spin-coated *Δ*-EuL PMMA films on glass with *Δ*-EuL in MeCN over the applied band-pass filter (BPF) range

BP filter	Glass substrate *g*_CPLP_				Solution state *g*_CPLP_	% *g*_CPLP_ reduction
	1	2	3	4		
BPF594/10	+0.10	+0.10	+0.11	+0.11	+0.21	50%
BPF610/10	−0.024	−0.024	−0.024	−0.024	−0.031	30%

To test this proposal, the glass substrate was covered with black matt tape, which is non-reflective in the wavelength of interest, non-emissive under 365 nm irradiation (SI Fig. 9) and not soluble in DCM. The same solution of *Δ*-EuL was then used to prepare 6 spin-coated PMMA films for CPLP (SI Fig. S12).

Similarly to the films spin-coated on glass, the obtained *g*_CPLP_ values (Table 2) were consistent across the 6 films using both BPFs which suggested high reproducibility of the method. The change of substrate increased the *g*_CPLP_ for the Δ*J* = 1 (BPF594/10); however, they are slightly lower (20%) compared to the solution. Similarly to that, the *g*_CPLP_ for the Δ*J* = 2 increased (BPF610/10) to become essentially equal to its *g*_lum_ equivalent.

**Table 2:** Comparison of *g*_CPLP_ for 6 sequentially spin-coated *Δ*-EuL PMMA films on glass and *Δ*-EuL in MeCN over the applied band-pass filter (BPF) range

BP filter	Black tape substrate *g*_CPLP_						Solution state *g*_CPLP_	% *g*_CPLP_ reduction
	1	2	3	4	5	6		
BPF594/10	0.17	0.17	0.16	0.17	0.17	0.16	0.21	20%
BPF610/10	−0.038	−0.039	−0.037	−0.041	−0.037	−0.038	−0.031	—

**Fig. 5:**
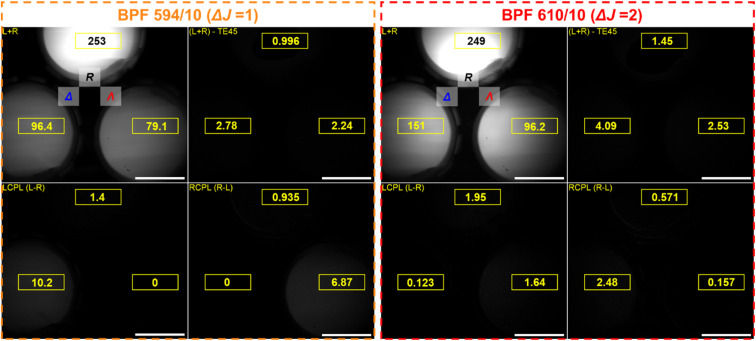
2 sets (using BPF594/10 and BPF610/10) of 4 CPLP images: (L + R, (L + R)-TE45, L − R, R − L) of the Δ (bottom left), Λ (bottom right) and racemic (R) (centre top) samples of EuL under 365 nm excitation with the regions of interest for *g*_CPLP_ and their average intensities shown in yellow. Scale bar = 5 mm.

In order to confirm the substrate dependence of the *g*_CPLP_, the experiment was repeated with solution state EuL. Black non-reflective in the wavelength region of interest non-emissive plastic caps (SI Fig. S9) were filled with *Δ*-EuL, *Λ*-EuL and racemic EuL solutions in acetonitrile of equal concentration and placed within a single frame for CPLP and EDCC (Fig. 5). The relevant areas of the calculated images were then used to calculate *g*_CPLP_ values of 0.21 and −0.17 for *Δ*-EuL and *Λ*-EuL respectively when using the BPF594/10 and ± 0.031 for the BPF610/10. This correlated with the *g*_lum_ values within the experimental error for dissymmetry factor of around ± 0.02 (∼11%) for the Δ*J* = 1 possessing brightest CPL and around ± 0.003 (∼9%) for Δ*J* = 2 when reflection is minimal. The racemate did not produce a significant intensity in either L − R or R − L images as expected.

The experimental error associated with CPLP could arise from the photoexcitation of the stage not being perfectly homogenous leading to unequal spatial excitation of the sample. As a result, certain pixels of the camera module might get oversaturated, serving as a source of error in image calculation. Moreover, the pixels within the module are not identical and might have slightly different sensitivity. The same applies to the imperfect alignment of the linear polarisers at each pixel. If distribution of such pixels is not random, the error is generated when a specific area of an image is selected for the average intensity measurement. Another source of error could be associated with inelastic scattering of the excitation light by the sample, making it fall into the detection wavelength range. Such light would likely be linearly polarised, and therefore L and R channels would be subjected to linear dichroism.

Since each enantiomer of EuL produces Δ*J* = 1 and Δ*J* = 2 of mutually opposite CPL sign, a single enantiomer results in detectable intensity in either (L − R) or (R − L) images depending on the BPF used. This makes EuL a unique security ink that simultaneously incorporates two CPL security layers. In contrast to this, most other reported CPL-active Eu^3+^ complexes could only produce detectable intensity in the wavelength region of the Δ*J* = 1 band.

**Fig. 6:**
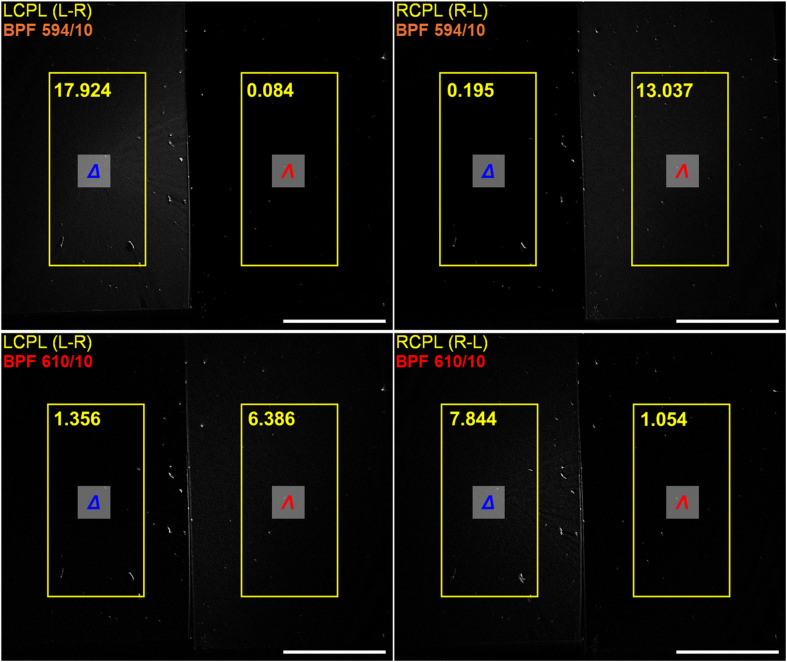
CPLP calculated images used for authentication of the proof-of-concept security tag containing *Δ*-EuL_1_ (left of each image) and *Λ*-EuL_1_ (right of each image) under 365 nm excitation with average intensities of the regions of interest shown in yellow scale bar = 5 mm.

As a proof of concept, two films spin coated on the matt black tape containing one enantiomer each were imaged together. For example, an authentic tag would contain *Δ*-EuL on the left-hand side and *Λ*-EuL on the right-hand side (right and left can be replaced with different parts of the security tag pattern). The left-hand side would then appear on the L − R image when using BPF594/10 (Δ*J* = 1) and R − L image when using BPF610/10 (Δ*J* = 2). Simultaneously, the right-hand side will show on the R − L image with the BPF610/10 and L − R image with the BPF594/10 (Fig. 6). Since sequentially produced films resulted in consistent *g*_CPLP_ values in both BPF594/10 and BPF610/10, the tag can be further secured by cross-checking the *g*_CPLP_ numerical values for each spatial region with the expected threshold. The whole sequence can be then automated and combined with other security layers as chromatic (colour), spectral (emission profile) and temporal (time-gating to cut off nanosecond scale emission of organic dyes present, lifetime of the both *Δ-* and *Λ-*EuL in PMMA films were measured as 0.8 ± 0.1 ms – see SI Fig. 14) that are already present in banknotes and identification documents.^1^

During prolonged experiments using CW excitation in excess of 1 hour, using our commercially available 365 nm UV LED irradiation source (1 W total power, collimated and focused to a *d* = 1 cm circle) we have not recorded any photodegradation and loss of CPL intensity of the studied films. These observations of photostability are based on camera exposure time and CPB being constant and unaltered throughout the studies.

## Conclusion

A novel bright CPL-active quasi*-C*_3_ symmetric EuL complex containing all carboxylate donor groups was prepared, chirally separated and photophysically characterised. EuL was exceptionally bright due to high values of the quantum yield (45%) and molar extinction coefficient (77 000 M^−1^ cm^−1^) while its near 365 nm maximal absorbance suggested optimal excitation by commercially available 365 nm UV LEDs. Invisible to the unaided naked eye, their large *pseudo*-Stokes’ shift allowed for absorption in the UV and emission in the visible regions. Emission lifetime on the millisecond time scale allows for temporal separation by time gating out nanosecond time-scale emission of applied organic dyes. Both Δ*J* = 1 and Δ*J* = 2 transitions demonstrated strong monosign CPL with CPB_max_ values of 307 mol^−1^ cm^−1^ at 596 nm and 590 mol^−1^ cm^−1^ at 607 nm respectively, rendering it to be best known candidate up to date for CPL security ink so far. The CPB_*i*_ was also calculated for the 594 ± 5 nm and 610 ± 5 nm single sign regions selected by BPFs to confirm the suitability of both transitions for authentication by CPLP. EuL was embedded into solid-state PMMA spin-coated thin films for a Proof of Concept (POC) security tag. Both enantiomers were successfully authenticated *via* CPLP and EDCC where dissymmetry of both Δ*J* = 1 and Δ*J* = 2 transitions was rapidly detected. The recorded dissymmetry was also quantified using the newly introduced term *g*_CPLP_ and the results compared to the average *g*_lum_ values recorded by the PEM-CPL spectrometer in the same wavelength regions. The *g*_CPLP_ values calculated from the EDCC images of solutions of EuL enantiomers were in good agreement with the *g*_lum_ values; however, the *g*_CPLP_ was significantly reduced in PMMA films on a glass substrate. The proposed hypothesis of dissymmetry reduction *via* reflection induced CPL sign cancellation from substrate was confirmed by replacing glass with black non-reflective tape that recovered the magnitude of *g*_CPLP_. The *g*_CPLP_ values remained consistent across multiple sequentially spin-coated films, suggesting high repeatability and consistent CPL security feature.

Spurred on from the reliability of *g*_CPLP_ as a performance indicator for CPL active chiral emitters when measured using our polarisation sensitive CPLP camera,^18^ as a future direction we propose to use blends of enantiopure Ln(iii)-complexes utilising CPL-fingerprint engineering resulting in the 2nd generation of more sophisticated CPL-CSIs (Chameleon Security Inks). The sign, and importantly the recorded numerical dissymmetry factor (g_CPLP_*via* CPLP) of these blended dyes, can be compared to an expected value during authentication. This will take security to another level, as the same exact blend of enantiopure (*Δ* and *Λ*) Ln(iii) complexes and their precise enantiomeric ratios would need to be replicated to counterfeit such security inks.

## Statistics and reproducibility

Where instruments incorporating a scanning monochromator have been used (absorption, emission, and excitation spectra) each sample has been recorded and averaged as triplicate measurements. Spectra, where CCD detectors have been employed, have been measured as an average of one hundred to a thousand spectra on triplicate samples according to the protocol detailed in the SI Section.

## Author contributions

AK: synthesised EuL complex, performed all photophysical measurements, assisted in chiral HPLC separation, contributed to the concept of CPLP and LED carousel design drafted and edited the manuscript. DJB: performed all 2-photon and quantum yield studies and edited the manuscript. AC: performed chiral HPLC analysis and separation. RP: secured project funding, conceptualised CPLP and associated detection methodology, designed the LED carousel, performed independent validation experiments, drafted and edited the manuscript.

## Conflicts of interest

R. P. is inventor on patent WO2016174395A1: light detecting apparatus for simultaneously detecting left-and right-handed circularly polarised light. R. P., D. J. B. and A. K. are inventors on patent application P408669.GB.01: Apparatus and method for detecting circularly polarised light. There are no other competing interests.

## Supplementary Material

SC-OLF-D5SC05303J-s001

## Data Availability

All data generated and analysed during this study including spectra and drawings are available from the corresponding author upon request. Code availability: custom codes written and developed and used during this study are available from the corresponding author upon request on a collaboration basis. Supplementary information (SI) is available. See DOI: https://doi.org/10.1039/d5sc05303j.

## References

[cit1] MacKenzie L. E., Pal R. (2020). Nat. Rev. Chem..

[cit2] Frawley A. T., Pal R., Parker D. (2016). Chem. Commun..

[cit3] Kitagawa Y., Wada S., Islam M. D. J., Saita K., Gon M., Fushimi K., Tanaka K., Maeda S., Hasegawa Y. (2020). Commun. Chem..

[cit4] De Rosa D. F., Starck M., Parker D., Pal R. (2024). Chem.–Eur. J..

[cit5] Hardwick B., Jackson W., Wilson G., Mau A. W. H. (2001). Adv. Mater..

[cit6] Carro-Temboury M. R., Arppe R., Vosch T., Sørensen T. J. (2018). Sci. Adv..

[cit7] Prime E. L., Solomon D. H. (2010). Angew. Chem., Int. Ed..

[cit8] Baek S., Choi E., Baek Y., Lee C. (2018). Digital Signal Process..

[cit9] Singh A. K., Singh S., Gupta B. K. (2018). ACS Appl. Mater. Interfaces.

[cit10] Kumar P., Dwivedi J., Gupta B. K. (2014). J. Mater. Chem. C.

[cit11] Sonnex E., Almond M. J., Baum J. V., Bond J. W. (2014). Spectrochim. Acta, Part A.

[cit12] Arppe R., Sørensen T. J. (2017). Nat. Rev. Chem..

[cit13] Imperio E., Calò E., Valli L., Giancane G. (2015). Vib. Spectrosc..

[cit14] Gariup M., Piskorski J. (2019). Int. J. Crit. Infrastruct. Prot..

[cit15] Lancaster I. M. and Mitchell A., in Optical Document Security, ed. R. L. Van Renesse, Artech House, Norwood, San Jose, CA, 3rd edn, 2004, p. 34

[cit16] Zinna F., Resta C., Abbate S., Castiglioni E., Longhi G., Mineo P., Di Bari L. (2015). Chem. Commun..

[cit17] Arrico L., Di Bari L., Zinna F. (2021). Chem.–Eur. J..

[cit18] De Rosa D. F., Stachelek P., Black D. J., Pal R. (2023). Nat. Commun..

[cit19] Soulié M., Latzko F., Bourrier E., Placide V., Butler S. J., Pal R., Walton J. W., Baldeck P. L., Le Guennic B., Andraud C., Zwier J. M., Lamarque L., Parker D., Maury O. (2014). Chem. -Eur. J..

[cit20] Evans N. H., Carr R., Delbianco M., Pal R., Yufit D. S., Parker D. (2013). Dalton Trans..

[cit21] D’Aléo A., Picot A., Beeby A., Gareth Williams J. A., Le Guennic B., Andraud C., Maury O. (2008). Inorg. Chem..

[cit22] Butler S. J., Lamarque L., Pal R., Parker D. (2014). Chem. Sci..

[cit23] Butler S. J., Delbianco M., Lamarque L., McMahon B. K., Neil E. R., Pal R., Parker D., Walton J. W., Zwier J. M. (2015). Dalton Trans..

[cit24] Neil E. R., Funk A. M., Yufit D. S., Parker D. (2014). Dalton Trans..

[cit25] Butler S. J., Delbianco M., Evans N. H., Frawley A. T., Pal R., Parker D., Puckrin R. S., Yufit D. S. (2014). Dalton Trans..

[cit26] Starck M., Fradgley J. D., De Rosa D. F., Batsanov A. S., Papa M., Taylor M. J., Lovett J. E., Lutter J. C., Allen M. J., Parker D. (2021). Chem.–Eur. J..

[cit27] Walton J. W., Bourdolle A., Butler S. J., Soulie M., Delbianco M., McMahon B. K., Pal R., Puschmann H., Zwier J. M., Lamarque L., Maury O., Andraud C., Parker D. (2013). Chem. Commun..

[cit28] Stachelek P., MacKenzie L., Parker D., Pal R. (2022). Nat. Commun..

[cit29] Frawley A. T. , Highly emissive chiral lanthanide(III) complexes for labelling and imaging, Doctoral thesis, Durham University, 2017

[cit30] Delbianco M., Lamarque L., Parker D. (2014). Org. Biomol. Chem..

[cit31] Latvaa M., Takalob H., Mukkala V.-M., Rodriguez-Ubisd J. C., Kankarea J. (1997). J. Lumin..

[cit32] Lakowicz J. R. and Masters B. R., Principles of Fluorescence Spectroscopy, 3rd edn, 2008, vol. 13

[cit33] Walton J. W., Carr R., Evans N. H., Funk A. M., Kenwright A. M., Parker D., Yufit D. S., Botta M., De Pinto S., Wong K.-L. (2012). Inorg. Chem..

[cit34] Starck M., MacKenzie L. E., Batsanov A. S., Parker D., Pal R. (2019). Chem. Commun..

[cit35] Thakur D., Vaidyanathan S. (2025). J. Mater. Chem. C.

[cit36] Walton J. W., Bari L. D., Parker D., Pescitelli G., Puschmann H., Yufit D. S. (2011). Chem. Commun..

[cit37] Petoud S., Muller G., Moore E. G., Xu J., Sokolnicki J., Riehl J. P., Le U. N., Cohen S. M., Raymond K. N. (2007). J. Am. Chem. Soc..

[cit38] Kumar J., Marydasan B., Nakashima T., Kawai T., Yuasa J. (2016). Chem. Commun..

[cit39] Zhou Y., Li H., Zhu T., Gao T., Yan P. (2019). J. Am. Chem. Soc..

[cit40] Schindelin J., Arganda-Carreras I., Frise E., Kaynig V., Longair M., Pietzsch T., Preibisch S., Rueden C., Saalfeld S., Schmid B., Tinevez J.-Y., White D. J., Hartenstein V., Eliceiri K., Tomancak P., Cardona A. (2012). Nat. Methods.

