# Supplementary material for: Complete stereochemical control to unlock monosign circularly polarised luminescence with superior circularly polarised brightness for chameleon security inks
Source: Chem Sci. 2026 Jan 12;17(9):4649–58. doi: 10.1039/d5sc05303j (PMC12794813; doi:10.1039/d5sc05303j)
Supplement: SC-017-D5SC05303J-s001 [file SC-017-D5SC05303J-s001.pdf]

Supplementary Information (SI) for Chemical Science.
This journal is © The Royal Society of Chemistry 2026

Complete Stereochemical Control to Unlock Monosign Circularly Polarised Luminescence with Superior Circularly Polarised Brightness for Chameleon Security Inks

Artemijs Krimovs,[1] Dominic J. Black[1], Aileen Congreve[1] and Robert Pal[1]*

[1]Department of Chemistry, Durham University, South Road, Durham, DH1 3LE, UK

*robert.pal@durham.ac.uk

Supplementary Information

Table of Contents

1. General procedures 2

2. Photophysical measurements 3

    2.1. Absorption, emission and lifetime measurements 3

    2.2.CPL spectroscopy 3

    2.3. Circularly polarised luminescence photography (CPLP) and enantioselective differential chiral contrast (EDCC) 4

3. Chiral HPLC and Racemisation Studies. 7

4. Spin coating and atomic force microscopy 9

5. Other Supplementary Figures 10

6. Synthetic Procedures 13

Supplementary References 32

1. General procedures

All starting materials are commercially available and distributed by Merck. Analytical and HPLC grade solvents were used and degassed using freeze-pump-thaw or cycling where appropriate.

Silica gel plates on aluminium support (0.2 mm thick, 60 F254, Merck) were used in thin layer chromatography (TLC) and resolved under UV radiation source at 254 or 365 nm. Flash column chromatography was manually prepared using silica gel (60 (230-400 mesh, Flourochem).

Preparative reverse-phase HPLC was performed using a Shimadzu module comprising of a Vacuum Degasser (DGU-20AR5), a Prominence Liquid Chromatography pump (LC-20AP), a Prominence UV-Vis Detector (SPD-20A) and a Communications Bus Module (CBM-20A). Sample separation was performed using a preparative XBridge C_{18} OBD column (19 × 100 mm, 5 µm, flow rate = 17 cm^3 min^{-1}) with fractions collected manually. The solvent system was a mixture of H_2O and MeCN, where the proportion of the two solvents was varied in time(Table 1).

Table 1: proportion of H_2O and MeCN or MeOH solvents in time-dependent gradient elution.

Step	Time/ mins	H_2O fraction	MeCN fraction
0	0	0.9	0.1
1	4	0.9	0.1
2	14	0	1
3	19	0	1
4	22	0.9	0.1

^1H and ^{13}C NMR was performed on A Bruker Advance-400 (^1H at 400.06 MHz and ^{13}C at 100.61 MHz), a Mercury 400 (^1H at 399.95 MHz), a Varian VNMRS-600 (^1H at 599.67 MHz and ^{13}C at 150.79 MHz) or a Varian VNMRS-700 (^1H at 699.73 MHz and ^{13}C at 175.95 MHz) using deuterated chloroform (Merck). Chemical shifts and J-couplings were reported in ppm and Hz respectively.

Electrospray ionisation mass spectrometry was performed on a TQD mass spectrometer containing an Aquity UPLC system and an electrospray ionisation source. Accurate mass determination was performed on an LCT Premier XE mass spectrometer or a QToF Premier Mass spectrometer. All mass spectrometers were equipped with an Aquity photodiode array detector (Waters Ltd., UK).

2. Photophysical measurements

2.1. Absorption, emission and lifetime measurements

All solution state optical analyses were carried out in quartz cuvettes with a path length of 1.0000 cm. Solid state (PMMA) samples were recorded using a custom-built holder. UV-Vis absorption was measured on an ATI Unicam UV-Vis spectrometer (Model UV2) using Vision software (version 3.33). Emission experiments were performed on an ISA Jobin-Yvon Spex Fluorolog-3 luminescence spectrometer using DataMax software (version 2.2.10). Lifetime was measured with a Perkin Elmer LS55 spectrometer using FL Winlab software either in solution state (using a range of solvents (Main text Figure 2D) or neat PMMA films (Figure S14). All emission experiments were performed at 1 μM solution concentration. Absolute photoluminescent quantum yields were determined using a Horiba FluoroMax Plus fitted with a QuantaPhi-2 integrating sphere in solution state (MeCN). All samples were recorded at 1 μM in a 1 cm by 1 cm path length quartz cuvette with four clear sides. Excitation and emission slits were fixed at 2 nm with neutral density filters (ThorLabs, NEK01S) used at the excitation aperture to maintain a signal of approximately 10^6 cps at the excitation wavelength.

2.2.CPL spectroscopy

CPL was measured with a home-built (modular) spectrometer.[1] The excitation source was a broad band (200 – 1000 nm) laser- driven light source EQ 99 (Elliot Scientific). The excitation wavelength was selected by feeding the broadband light into an Acton SP-2155 monochromator (Princeton Instruments); the collimated light was focused into the sample cell (1 cm quartz cuvette). Sample PL emission was collected perpendicular to the excitation direction with a lens (f = 150 mm). The emission was fed through a photoelastic modulator (PEM) (Hinds Series II/FS42AA) and through a linear sheet polariser (Comar). The light was then focused into a second scanning monochromator (Acton SP2155) and subsequently on to a photomultiplier tube (PMT) (Hamamatsu H10723 series). The detection of the CPL signal was achieved using the field modulation lock-in technique. The electronic signal from the PMT was fed into a lock-in amplifier (Hinds Instruments SignaLoc Model 2100). The reference signal for the lock-in detection was provided by the PEM control unit. The monochromators, PEM control unit and lock-in amplifier were interfaced to a desktop PC and controlled by a custom-written LabVIEW graphic user interface. The lock-in amplifier provided two signals, an AC signal corresponding to (IL- IR) and a DC signal corresponding to (IL+ IR) after background subtraction. The emission dissymmetry factor was therefore readily obtained from the experimental data, as 2 AC/DC. Spectral calibration of the scanning monochromator was performed using a Hg-Ar calibration lamp (Ocean Optics, HG-2). A correction factor for the

wavelength dependence of the detection system was constructed using a calibrated lamp (Ocean Optics, HL-2000). The measured raw data was subsequently corrected using this correction factor. The validation of the CPL detection systems was achieved using light emitting diodes (LEDs) at various emission wavelengths. The LED was mounted in the sample holder and the light from the LED was fed through a broad band polarising filter and $\lambda/4$ plate (ThorLabs, LPVISE100-A and AQWP05M-600) to generate circularly polarised light. The emission spectra were recorded with 0.5 nm step size and the slits of the detection monochromator were set to a slit width corresponding to a spectral resolution of 0.25 nm. CPL spectra (as well as total emission spectra) were obtained through an averaging procedure of several scans.; all calculations were carried out using raw spectral data. Prior to all measurements, the $\lambda/4$ plate and a LED were used to set the phase of the lock-in amplifier correctly.

2.3. Circularly polarised luminescence photography (CPLP) and enantioselective differential chiral contrast (EDCC)

The CPLP handheld instrument for EDCC comprises of an externally triggered Kiralux® polarisation 5.0 Megapixel CMOS USB camera (CS505MUP1, Thorlabs). It is synchronised to a 365 nm LED (Nichia, 5.2V, 500mW) flash illuminator driven by an internal custom built signal generator module operating at master frequencies of 0.1, 1 or 10 Hz. This device has been constructed to be able to provide both a variable illumination (1 ms - 1 s) and image acquisition (0.027 ms – 14 s) sequence with a constant 20 μs time delay between the two pulses to allow discrimination of short lived organic (ns- μs) and long-lived lanthanide pseudo phosphorescence (ms). The chiroptical separator of the apparatus comprises of the built in pixel decoded wire grid polariser array complemented by a precisely aligned broad wavelengths (λ = 400 – 800 nm) quarter waveplate (QWP, Thorlabs AQWP05M-600), a machine vision (Navitar f = 25mm/F1.4) objective lens (interchangeable to a LWD f-50 mm/F2.8 lens and 0.63x video relay lens (Edmund Optics) for epifluorescence microscopy) and a selectable filter wheel containing high precision narrow band pass filters (such as Edmund optics BP546/10, BP589/10, BP594/10, and BP610/10) to achieve chromatic discrimination.

Camera control and image acquisition were performed with ThorLab's commercial camera software ThorCam™ or an adapted custom LabView code to facilitate time-resolved detection and controlled external camera triggering.[43] The camera was operated in quad view (Figure SI 20) where the 16-bit overlayed total image has been split up into four individual 8-bit images decoding each wire grid polariser state orientation (0, 90, 45 and -45 degree) captured image as a 2 x 2 array in one captured frame. These images were generated by area defined crop and paste individual image generation without pixel position reassignment. Such as the

previously reported EDCC using our recently developed CPL-LSCM, this one step resolution preserving (lossless compression) image processing is achieved using a custom written script (macro) in ImageJ (v1.53).[2] EDCC imaging was also built into our custom written macro 2.0 or can be post processed using ImageJ's built-in image calculator add-on software by subtracting one CPL channel from the other, and vice versa. The convention used herein is: left-handed enantioselective contrast = left CPL – right CPL; right-handed enantioselective contrast = right CPL – left CPL. Images were recorded with the camera's native 2448 x 2048 pixel field of view (FOV) without image cropping with total accumulated integration time varied from 20 ms to 1 s with 5 – 32 frame averaged sequences. The total integration time has been determined case by case by careful monitoring of maximum 8-bit pixel values to eliminate pixel saturation and achieve maximum overall image brightness for EDCC calculation.

The all-important 8-bit average pixel chiroptical contrast value calculations were facilitated by selecting and averaging equal size and shape arbitrary area portions of the sample. Due to the 2448 x 2024 pixel size of each recorded image total field of view (FOV), this arbitrary area has been kept at a constant size of 300 x 600 pixels region of interest (ROI). The average maximum 8-bit greyscale pixel intensity values were determined using ImageJ Analyse-Measure macro with mean ROI intensity value mode that employs a maximum average value ROI histogram methodology that is based on a standard Gaussian distribution profiling of the average intensity values. Due to the employed methodology and the averaging nature of image acquisition and ROI calculation, the limit of detection (error associated with) 8-bit greyscale contrast value is below 1% (< 3 average greyscale value/pixel on a 0 – 255 pixel intensity scale). This is determined using the total europium emission image and the selected arbitrary ROI area selection is then kept identical throughout the imaging sequence resulting in high precision chiroptical contrast calculations.

2.4. Two photon cross section determination

The cross-section (σ^2) is calculated according to established procedures:[3]

$$\sigma^2 = \frac{F_S}{\phi_S C_S} \times \frac{\phi_R C_R \sigma_R^2}{F_R} \#[1]$$

where *S* is sample, *R* is reference, ϕ is the total emission quantum yield of the EuL (ϕ_S), *C* is the concentration, *n* is the refractive 7 index and F_S (λ) and F_R (λ) are the integrated PL spectrum for the sample and reference, respectively. Additionally, the two-photon nature of the excitation process was proven by recording an excitation power dependence; the resulting line has a slope of two on a logarithmic scale.[4] The cross sections were calculated with reference to Rhodamine B in methanol.

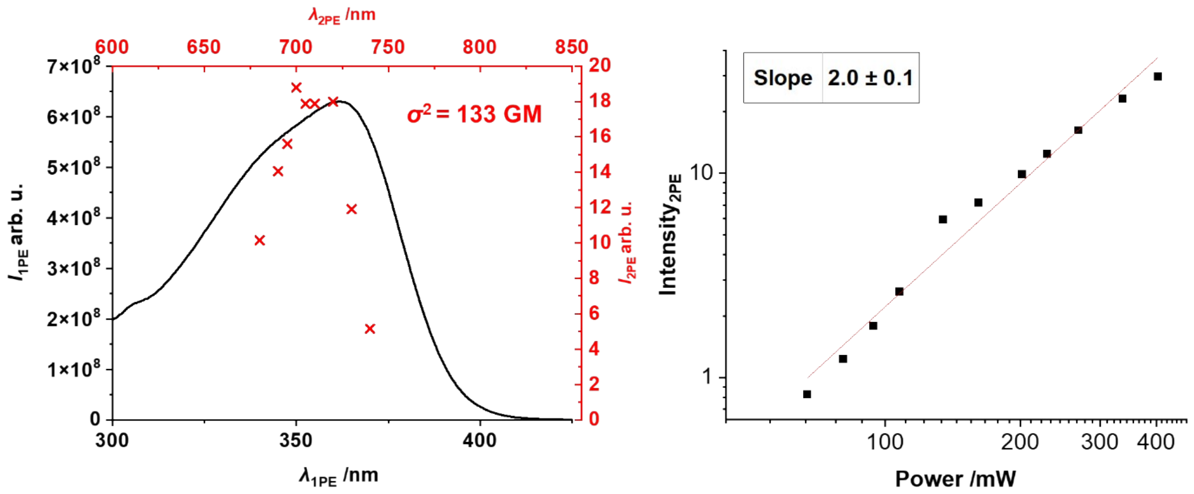

Figure S1: 1PE (black) and 2PE (red) spectra (λ_{em} = 615 nm) of EuL in MeCN (left) and integrated emission intensity plot against the laser excitation power (λ_{2PE} = 700 nm) on a logarithmic scale (right); GM = 10^{-50} cm^4 s $photon^{-1}$.

Two photon spectroscopy was performed using a tuneable femtosecond pulsed laser (680 – 1300 nm, Coherent Discovery TPC, 100 fs, 80 MHz) perpendicularly mounted to an Ocean Optics HR2000Pro (2048- pixel linear CCD Sony ILX5 chip, 200 μm slit, H3 grating, 350 – 850 nm spectral region) spectrometer.[5] The laser beam was focused onto the centre of the 1 cm path sample holder (Thor labs CVH100) by a dedicated ultrafast laser lens (Edmund Optics 11711, 50 mm focal length). The spectrometer has also been equipped with a perpendicularly mounted 365 nm LED (nichia, 1W) and been operated using a modified version of the above-mentioned custom time resolved detection and accumulation algorithm written in Labview2013 program. In order to eliminate unwanted artefacts associated with stray light from MP excitation the spectrometer have been equipped with a rotating filter wheel (Thor Labs, CFW6) housing an LP420 (Comar Optics, for 365 nm UVLED excitation) and SP650 and SP700 (Edmund Optics, 8472 and 8474 for MP excitation) filters.

3. Chiral HPLC and Racemisation Studies.

Chiral HPLC was performed using a Perkin Elmer Series 200 module consisting of a Perkin Elmer Series 200 pump, autosampler, UV-Vis detector and either CHIRALPAK-IE or IB N-5 columns (4.6 × 250 mm, flow rate = 2 ml min^{-1} at 40 °C). The solvent system consisted of EtOH/MeOH/TEA/TFA at 50/50/0.5/0.3 ratio; injection volume: 50 μL; sample concentration: 0.5 mg/ml in acetonitrile.

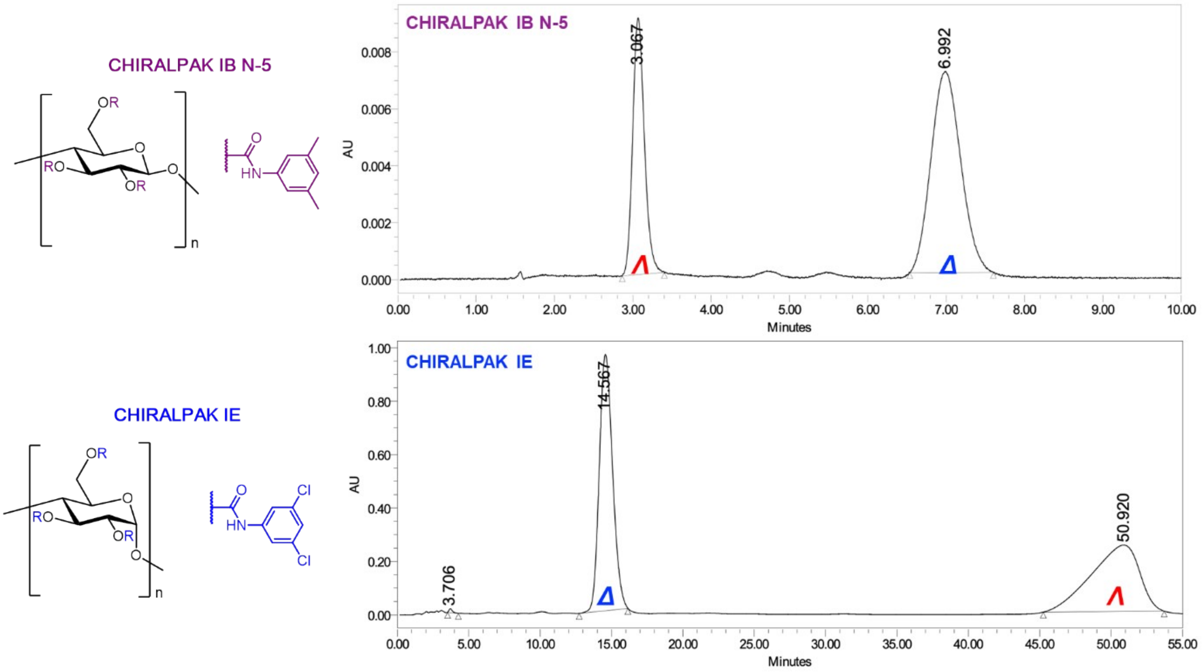

Figure S2: chiral high performance liquid chromatography (CHPLC) chromatogram (360 nm) for the chiral separation of the enantiomers of EuL using the CHIRALPAK IE and IB N-5 columns (stationary phase structures shown).

Solutions of *Δ*-EuL with ~0.5 aborbance at 360 nm were prepared in MeCN, MeOH, MeOD and 2-PrOH and then heated and stirred at 60 °C. 20 μL fractions of each solution was taken after every 25 hours and frozen for chiral HPLC analysis. The same was repeated for another solution of *Δ*-EuL in MeCN but without heating. The resulting solutions were analysed using CHIRALPAK IE column using the method above. The absorbance chromatograms recorded at 360 nm was then used to express the concentration of the enantiomers by integrating the area under the peaks. When racemisation of the enantiopure sample with 100 % enantiomeric excess of the *Λ* enantiomer is considered, an equilibrium is established:

$$\Lambda \underset{K_{-1}}{\overset{K_1}{\rightleftharpoons}} \Delta \qquad [2]$$

where the rates of interconversion for each enantiomer is assumed to be equal $k_1 = k_{-1} = k$. Therefore, the rate expression for racemisation can be written as

$$\frac{-d[\Lambda]}{dt} = k[\Lambda] - k[\Delta] = k([\Lambda] - [\Delta])\#[3]$$

$$\frac{1}{[\Lambda] - [\Delta]} d[\Lambda] =- kdt\#[4]$$

Since $[\Delta] = [\Lambda]_0 - [\Lambda]$, integration of the rate equation gives

$$\int_{[\Lambda]_0}^{[\Lambda]} \frac{1}{2[\Lambda] - [\Lambda]_0} d[\Lambda] = \int_{t_0}^{t} - k\ dt\#[5]$$

$$\frac{ln(2[\Lambda] - [\Lambda]_0) - ln(2[\Lambda]_0 - [\Lambda]_0)}{2} =- kt\#[6]$$

$$ln\left(\frac{[\Lambda]_0}{2[\Lambda] - [\Lambda]_0}\right) = 2kt\#[7]$$

which allows for determination of 2*k* by taking gradient of the plot of $ln\left(\frac{[\Lambda]_0}{2[\Lambda] - [\Lambda]_0}\right)$ against *t*.

The half-life for racemisation ($\tau_{1/2}$) is a time during which the enantiomeric excess of 50% is reached, which can be interpreted as a mixture containing 75% of the starting enantiomer and 25% of the other enantiomer which was formed by interconversion. Substitution of 1 and 0.75 into $[\Lambda]_0$ and $[\Lambda]$ respectively gives equation for the relationship between the experimentally obtained 2*k* and $\tau_{1/2}$.

$$2k\tau_{1/2} = ln\left(\frac{1}{2 \times 0.75 - 1}\right) = ln2\#[8]$$

$$\tau_{1/2} = \frac{ln2}{2k}\#[9]$$

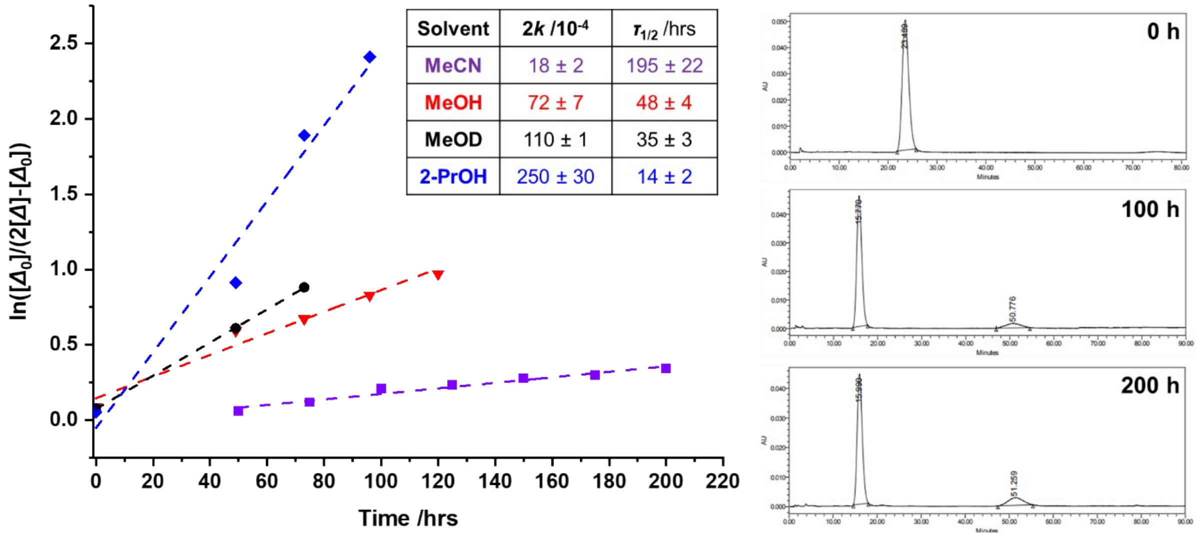

Solvent	$2k$ /10^{-4}	$\tau_{1/2}$ /hrs
MeCN	18 ± 2	195 ± 22
MeOH	72 ± 7	48 ± 4
MeOD	110 ± 1	35 ± 3
2-PrOH	250 ± 30	14 ± 2

Figure S3: ln($[\Delta_0]$/(2$[\Delta]$-$[\Delta_0]$)) against heating time at 60 °C in MeCN (purple), MeOH (red), MeOD (black) and 2-PrOH (blue) with obtained gradients (2*k*) and calculated racemisation half-life ($\tau_{1/2}$) where $[\Lambda]$ and $[\Lambda_0]$ are factual and initial concentrations of *Λ*-EuL respectively (left); three examples of 360 nm absorbance chromatograms for *Δ*-EuL heated in MeCN at 60 °C (right).

4. Spin coating and atomic force microscopy

Spin coating solutions contained 22 mg PMMA (average M_w ~15000 by gel permeation chromatography, density 1.18 g cm^{-3}) and ~17 µg of EuL (obtained by preparing 5 ml of 0.22 max absorbance solution and then evaporating the solvent) dissolved in 0.500 ml of DCM. This resulted in PMMA concentration of 44 mg ml^{-1} in DCM and 650 µM EuL concentration in PMMA. The solutions were spin coated on 1*1.5 cm cut glass microscope slides. To prepare films on the black electrical tape substrate, the tape was attached to the glass substrates and cut to size. Each film was prepared by casting 15 µl of the solution on the substrate spinning at 8000 rpm.

Atomic force microscopy was performed on an SPM SmartSPM™-1000 (AIST-NT) which consisted of the base and scanning head, both operated by an SPM controller. The base contained the sample metal holder that allowed for magnetic attachment of the SPM Specimen discs (15 mm, Agar Scientific) containing a sample (polymer film spin-coated on a microscope glass slide (manually cut to 1.5 × 1.0 cm) which was attached to the specimen discs using carbon tabs (9mm, Agar Scientific). The metal holder was attached to a digitally controlled (SmartSPM software) motorised positioning system allowing for translation of the sample holder vertically (18 mm range) and horizontally (5 × 5 mm). The scanning head comprised of a cantilever probe holder, laser and a four-sectional photodiode. The probe was a silicon tip with the 10 nm diameter and force constant of 0.02 - 0.77 N/m designed for contact-mode (NANOSENSORS™).

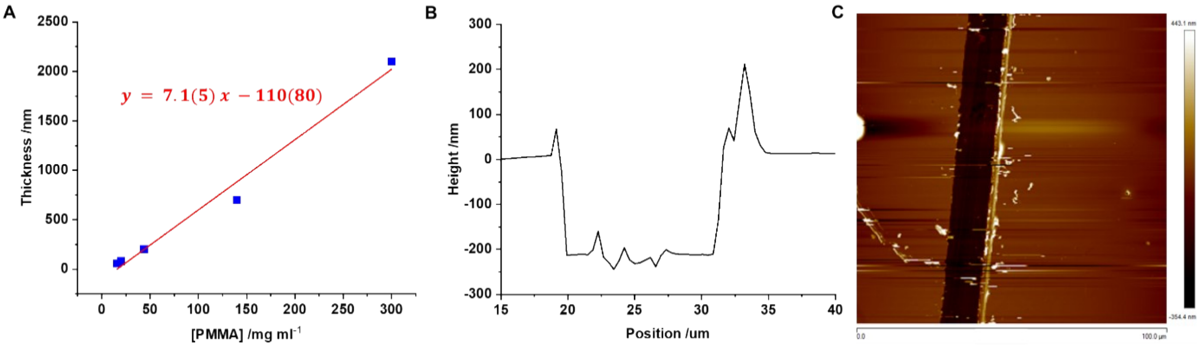

Figure S4: The established dependence of the PMMA concentration ([PMMA]) on the resulting film thickness spin-coated at 8000 rpm (A); the example of depth profile (B) and the corresponding AFM image (C) of an incision on a spin-coated PMMA film using 44 mg ml^{-1} [PMMA].

5. Other Supplementary Figures

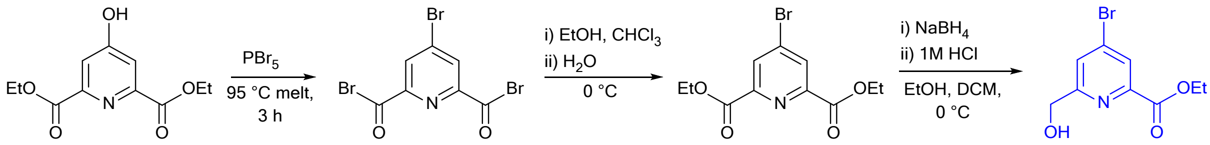

Figure S5: Synthesis of the ‘bottom’ of the sensitising chromophore.

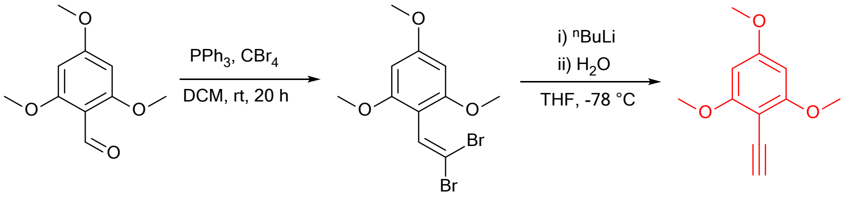

Figure S6: Synthesis of the ‘top’ of the sensitising chromophore.

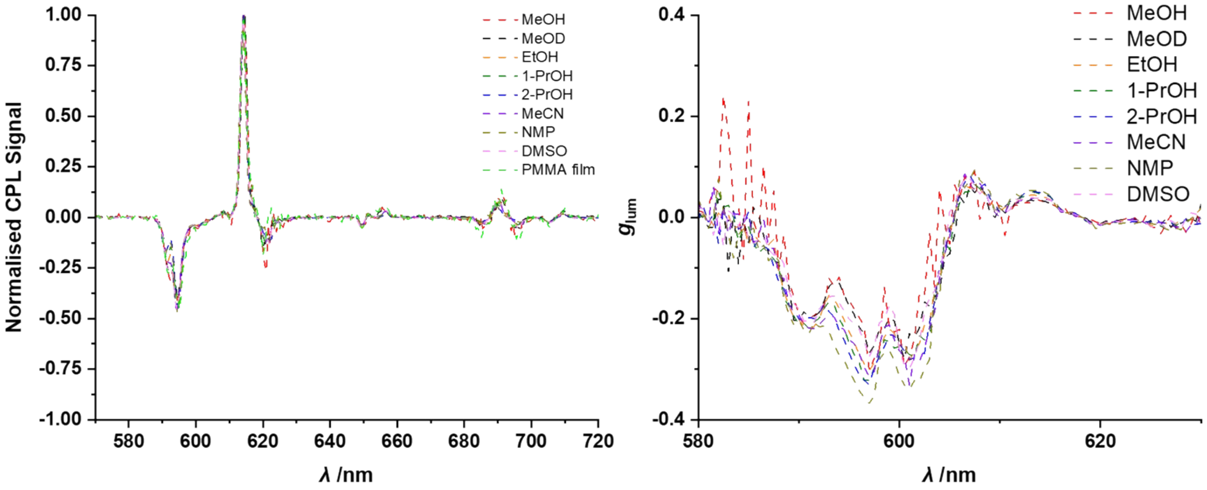

Figure S7: Normalised (0 to 1) CPL spectra of *Λ*-EuL in different solvents demonstrating the CPL profile independence on solvent (left) and the corresponding g_{lum} plots for the $\Delta J = 1$ and $\Delta J = 2$ transitions.

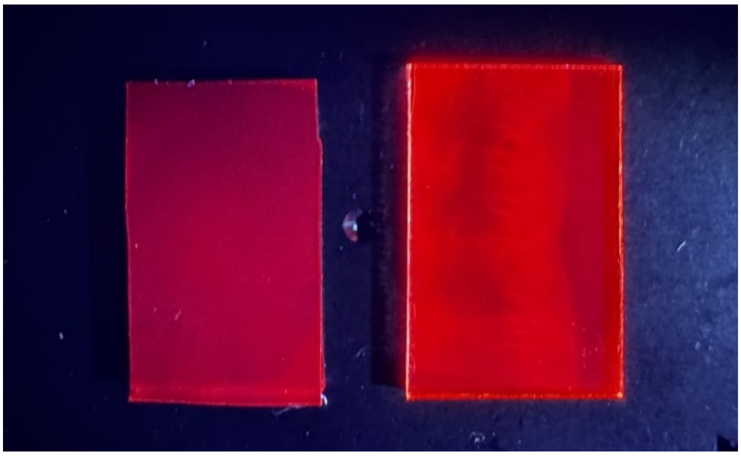

Figure S8: photograph of the *Δ*-EuL_1 containing PMMA films spin-coated on black electrical tape (left) and glass (right) under 365 nm UV irradiation.

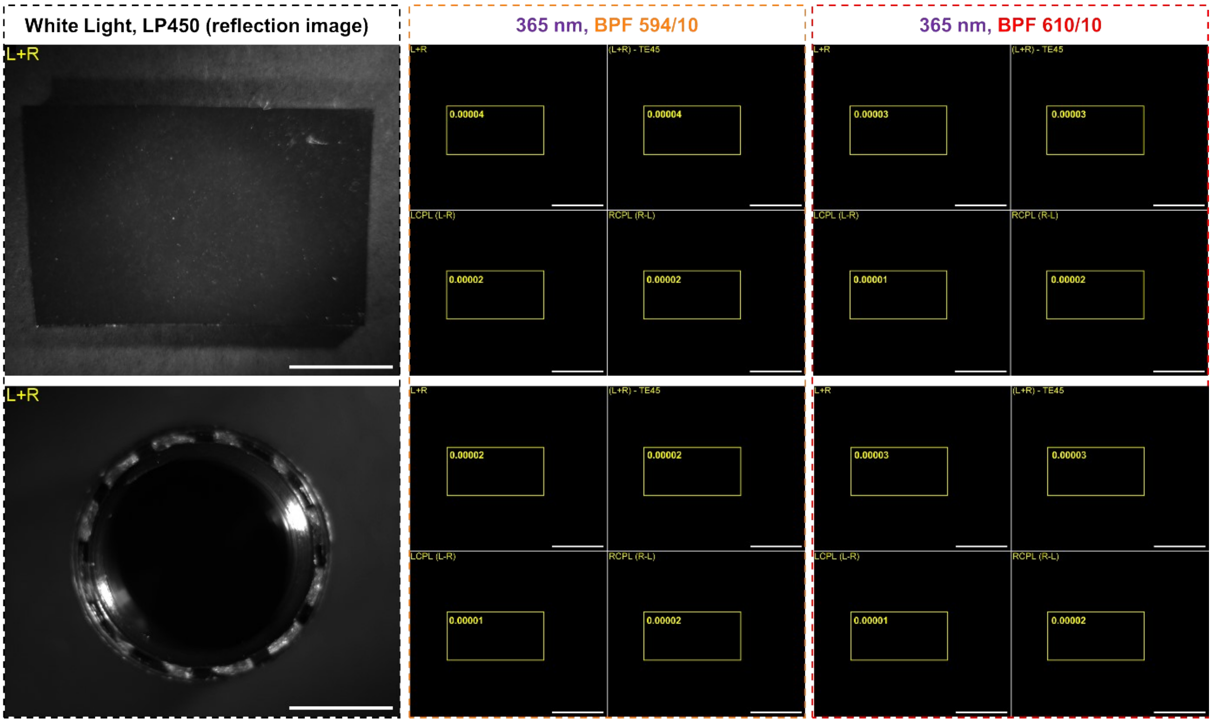

Figure S9: CPLP of black tape substrate (top) and black plastic cap (bottom) using BPF594/10 and BPF610/10 under 365 nm excitation with the regions of interest and their average intensities shown in yellow. The relevant L+R images under white light using 450 nm long pass filter (LP450); Scale bar = 5 mm.

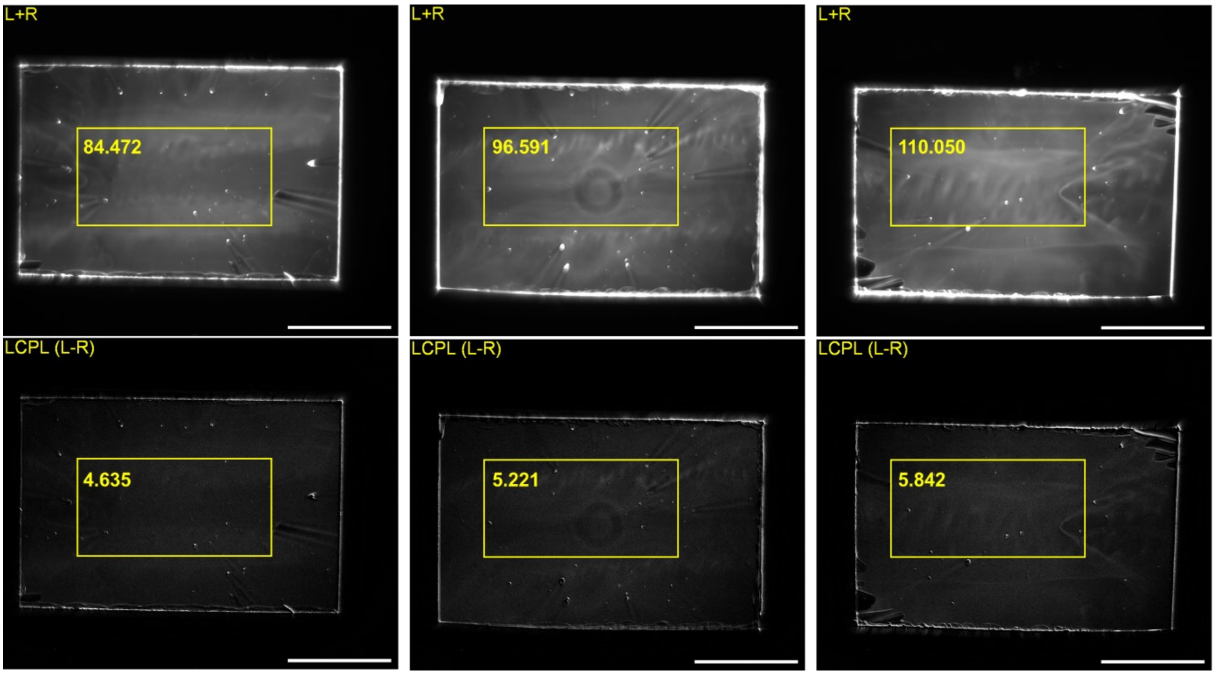

Figure S10: CPLP of three sequentially spin coated PMMA films containing *Δ*-EuL using BPF594/10 where L+R (top row) and L-R (bottom row) with the regions of interest for g_{CPLP} calculations and their average intensities shown in yellow. Scale bar = 5 mm. R-L and L-R images are 6 times contrast enhanced for display.

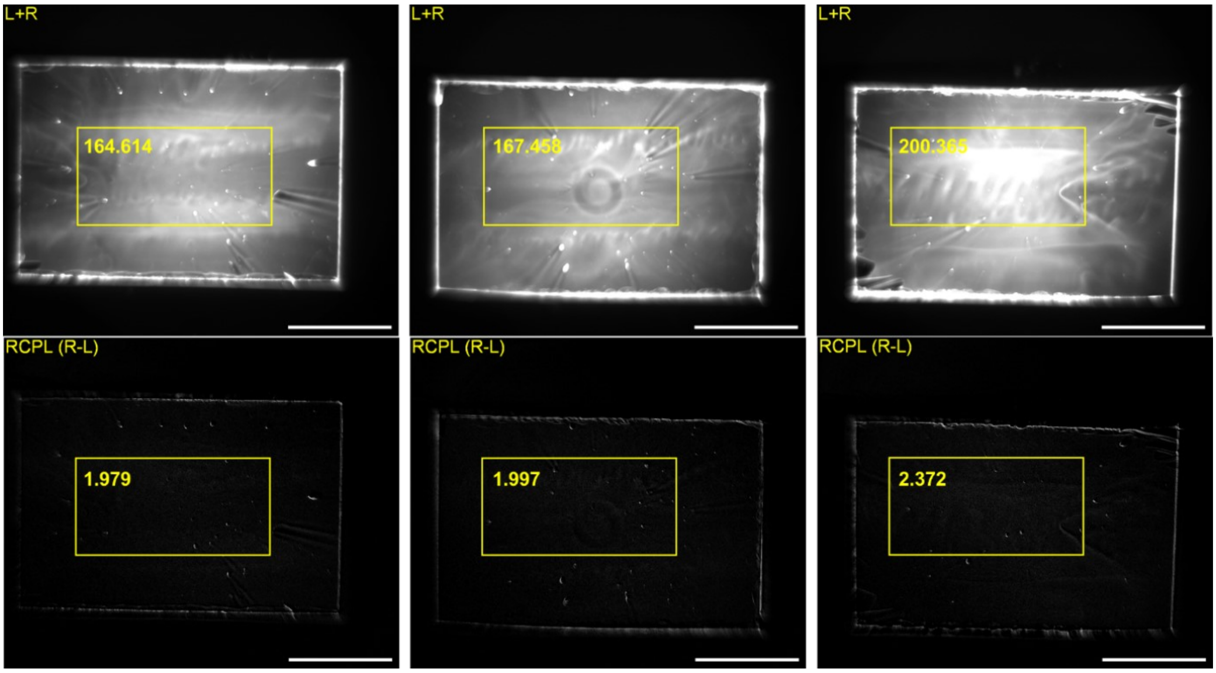

Figure S11: CPLP of three sequentially spin coated PMMA films containing *Δ*-EuL using BPF610/10 where L+R (top row) and R-L (bottom row) with the regions of interest for g_{CPLP} calculations and their average intensities shown in yellow. Scale bar = 5 mm. R-L and L-R images are 6 times contrast enhanced for display.

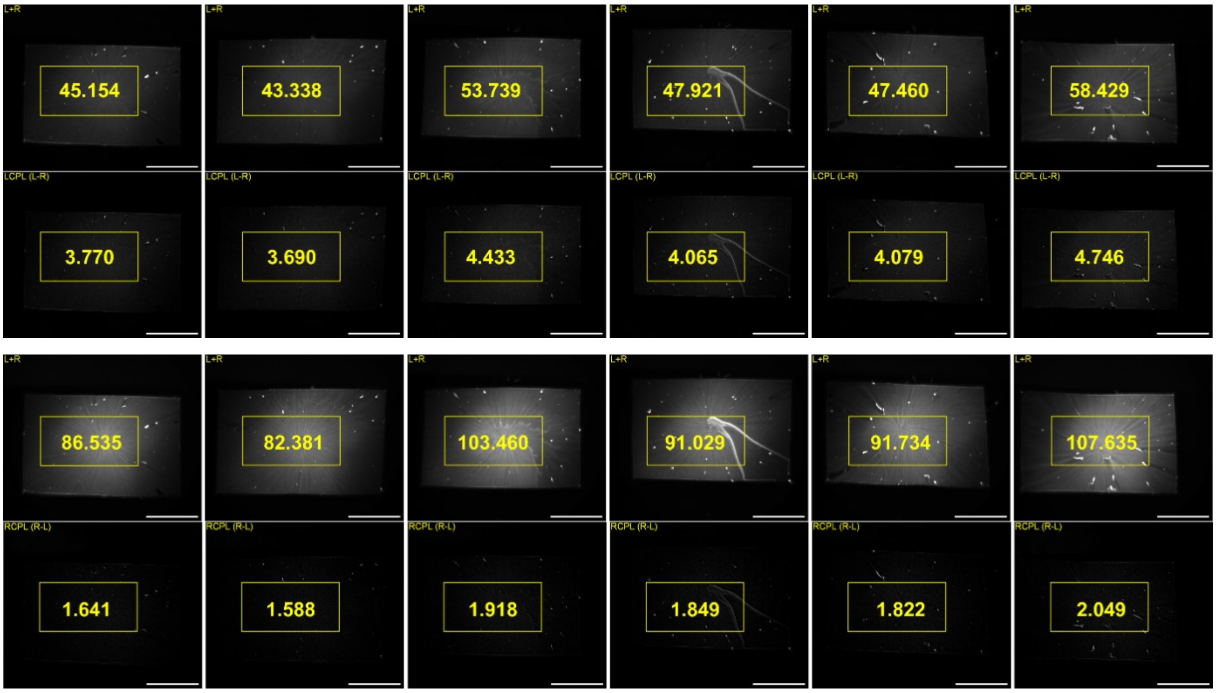

Figure S12: CPLP of six sequentially spin coated PMMA films containing *Δ*-EuL using BPF594/10 (top two rows) and BPF610/10 (bottom two rows) where L+R (1st and 3rd rowas) and L-R (2nd and 4th rows) with regions of interest for g_{CPLP} calculations and their average intensities shown in yellow. Scale bar = 5 mm. R-L and L-R images are 6 times contrast enhanced for display.

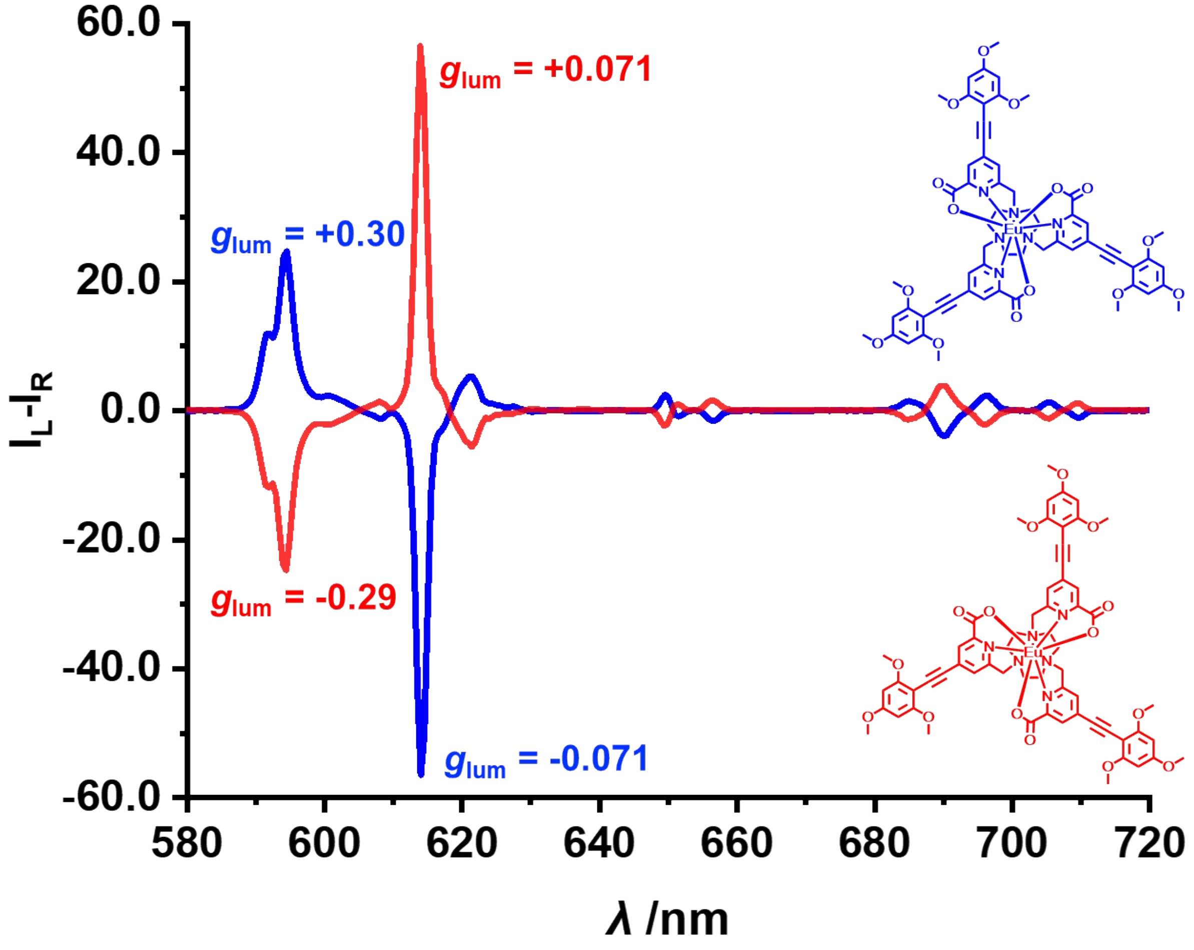

Figure S13: CPL spectra of *Δ*-EuL (blue) and *Λ*-EuL (red) (and their colour-coded structures) in acetonitrile, λ_{ex} = 360 nm, 5 averages.

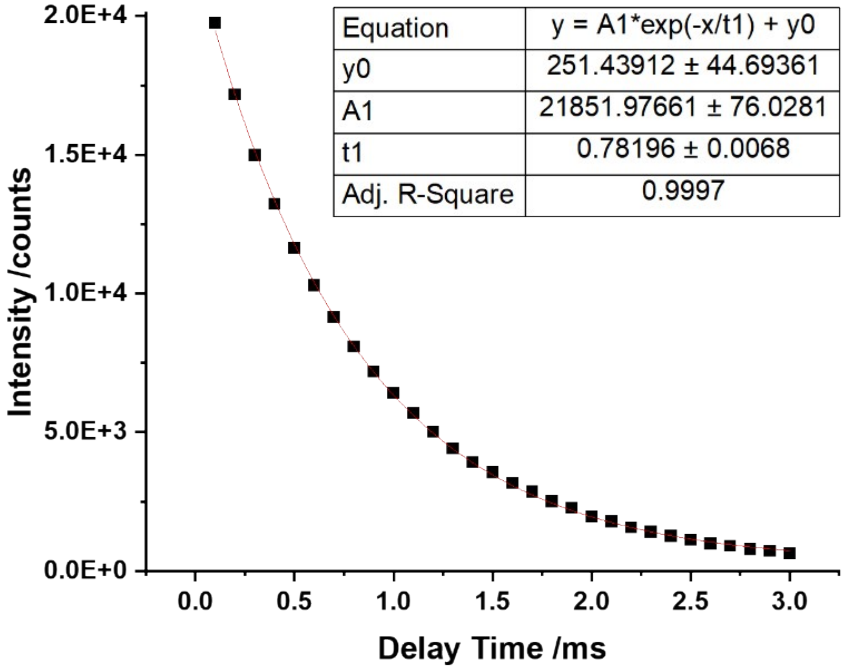

Equation	y = A1*exp(-x/t1) + y0
y0	251.43912 ± 44.69361
A1	21851.97661 ± 76.0281
t1	0.78196 ± 0.0068
Adj. R-Square	0.9997

Figure S14: Exponential decay fitted to a plot of lifetime data (0.1 ms steps) for a ~200 nm thick PMMA film spin-coated on a glass substrate containing EuL.

6. Synthetic Procedures

diethyl 4-bromopyridine-2,6-dicarboxylate

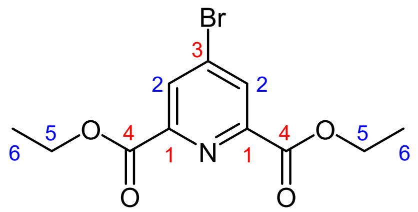

A solid mixture of chelidamic acid (1.64 g, 8.97 mmol, 1 eq.) and phosphorus pentabromide (11.6 g, 26.9 mmol, 3 eq.) was melted and heated (95 °C, 3 h). After that anhydrous chloroform (30 ml) was added, and the resulting hot mixture was filtered and cooled in an ice bath. Cold ethanol was then added drop-wise (20 ml), and the resulting solution was stirred (rt, 15 min). After that, the solvent was evaporated under the reduced pressure to yield brown oil. It was then dissolved in ice-cold water and stirred (0 °C, 1 h) to allow for precipitation of the crude. The resulting brown solid was dried and recrystallised from hexane to yield light-brown solid (1.23 g, 4.07 mmol, 45%);

^{1}H NMR (400 MHz, $CDCl_3$) δ 8.42 (2 H, s, H^2), 4.50 (4 H, q, $^3J_{H\text{-}H}$ 7.1, H^5), 1.46 (6 H, t, $^3J_{H\text{-}H}$ 7.4, H^6);

HRMS (ESI$^+$) *m/z* 302.0023 ($C_{11}H_{13}NO_4Br$ requires 302.0028).

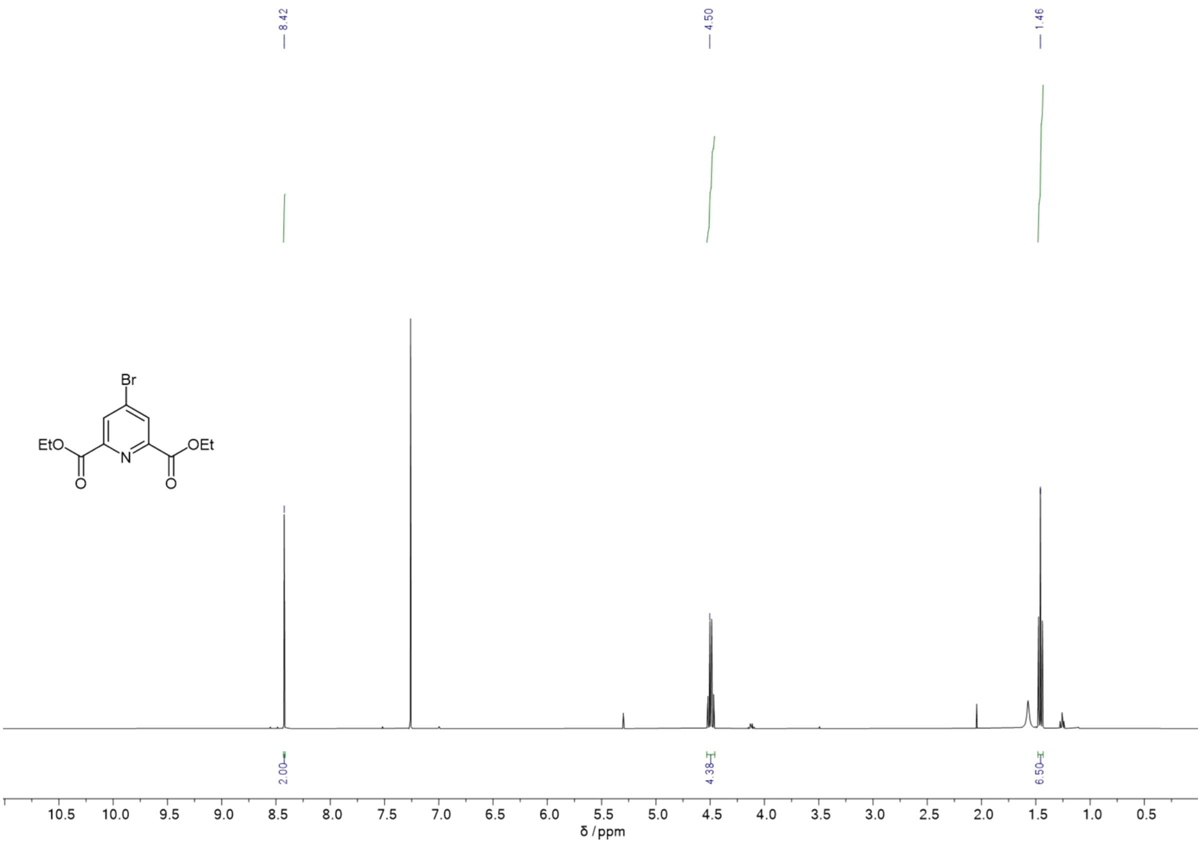

Figure S15: ^{1}H NMR (400 MHz, $CDCl_3$) spectrum of diethyl 4-bromopyridine-2,6-dicarboxylate.

Single Mass Analysis
Tolerance = 3.0 mDa / DBE: min = -1.5, max = 50.0
Element prediction: Off
Number of isotope peaks used for i-FIT = 5

Monoisotopic Mass, Even Electron Ions
976 formula(e) evaluated with 8 results within limits (up to 200 closest results for each mass)
Elements Used:
C: 0-100 H: 0-100 N: 0-20 O: 0-20 Br: 0-2

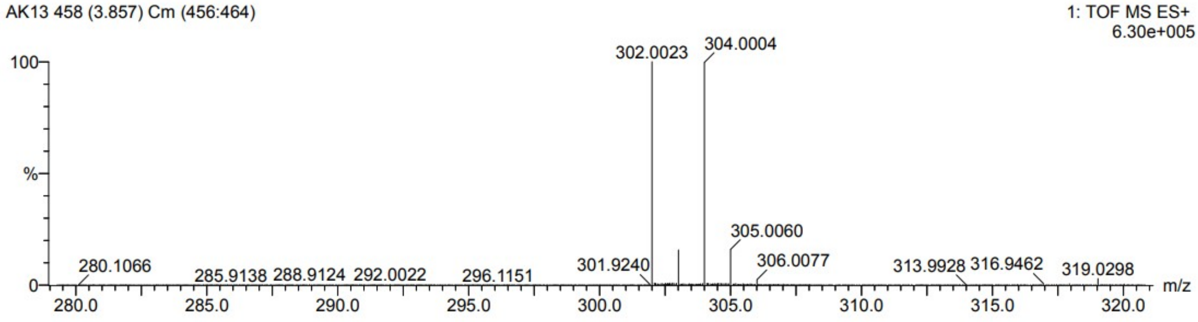

Minimum:				-1.5			
Maximum:		3.0	5.0	50.0			
Mass	Calc. Mass	mDa	PPM	DBE	i-FIT	i-FIT (Norm)	Formula
302.0023	302.0022	0.1	0.3	13.5	1447.5	21.2	C8 N9 O5
	302.0028	-0.5	-1.7	5.5	1427.2	0.9	C11 H13 N O4 Br

Figure S16: HRMS data for diethyl 4-bromopyridine-2,6-dicarboxylate.

ethyl 4-bromo-6-(hydroxymethyl)picolinate

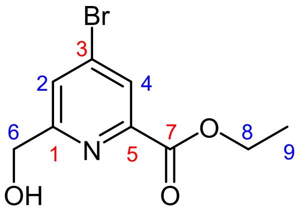

Diethyl 4-bromopyridine-2,6-dicarboxylate (1.23 g, 4.07 mmol, 1 eq.) was dissolved in a solution of dichloromethane (7 ml) and ethanol (9 ml) and cooled in an ice bath. After that, solid sodium borohydride (0.23 g, 6.11 mmol, 1.5 eq.) was slowly added and the resulting solution was stirred (0 °C, 4 h), while monitoring the reaction using TLC (silica, 3% methanol in dichloromethane). After that, a solution of 1 M hydrochloric acid (6 ml) was added, followed by water (25 ml) and dichloromethane (20 ml). The resulting aqueous layer was separated and washed with dichloromethane (10 ml × 3). After that, the combined organic layers were washed with water (10 ml × 3), dried over magnesium sulphate, filtered and concentrated under reduced pressure. The crude solution was then purified by column chromatography (silica, dichloromethane to 2.5% methanol in dichloromethane) to yield white solid (0.675 g, 2.60 mmol, 64%);

^{1}H NMR (400 MHz, $CDCl_3$) δ 8.17 (1 H, d, $^4J_{H-H}$ 1.83, H^4), 7.73 (1 H, d, $^4J_{H-H}$ 1.83, H^2), 4.84 (2 H, s, H^6), 4.46 (2 H, q, $^3J_{H-H}$ 6.9, H^8), 1.43 (6 H, t, $^3J_{H-H}$ 7.1, H^9);

LCMS (ESI$^+$) *m/z* 261 [M+H]$^+$.

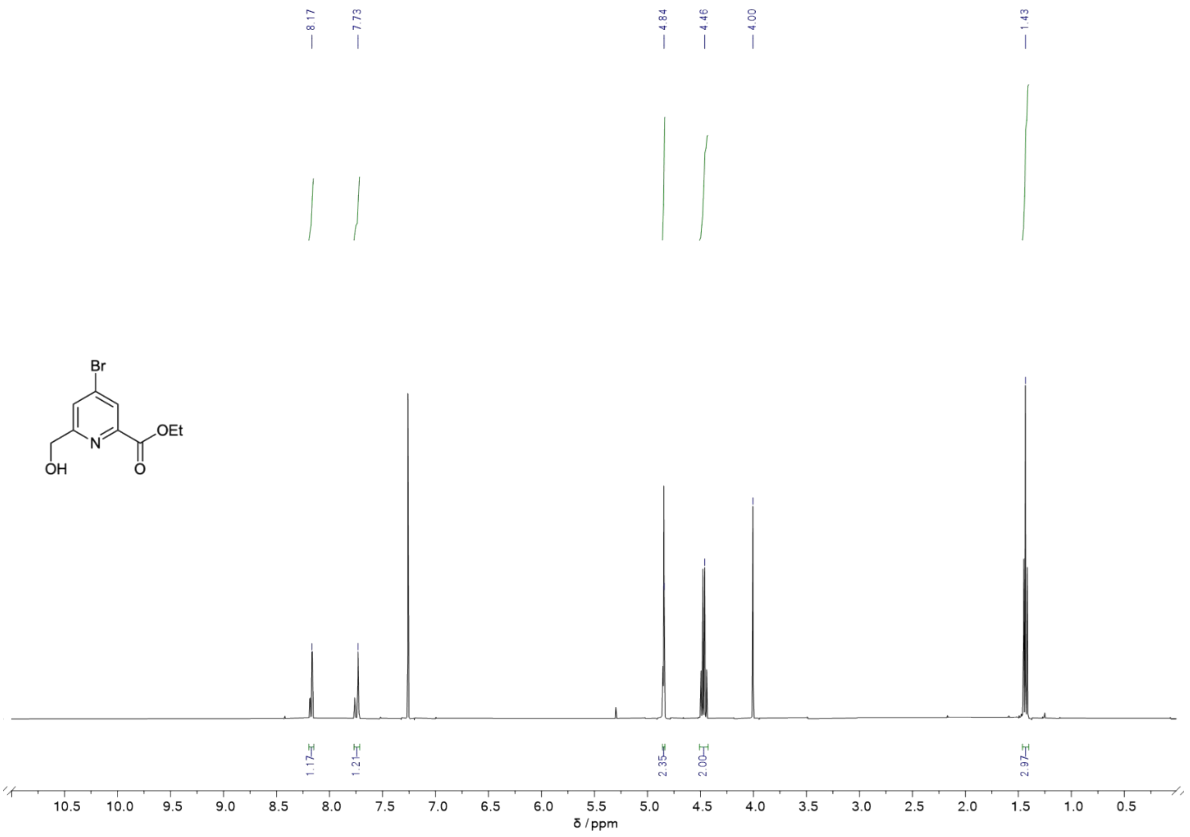

Figure S17: ^{1}H NMR (400 MHz, $CDCl_3$) spectrum of ethyl 4-bromo-6-(hydroxymethyl)picolinate.

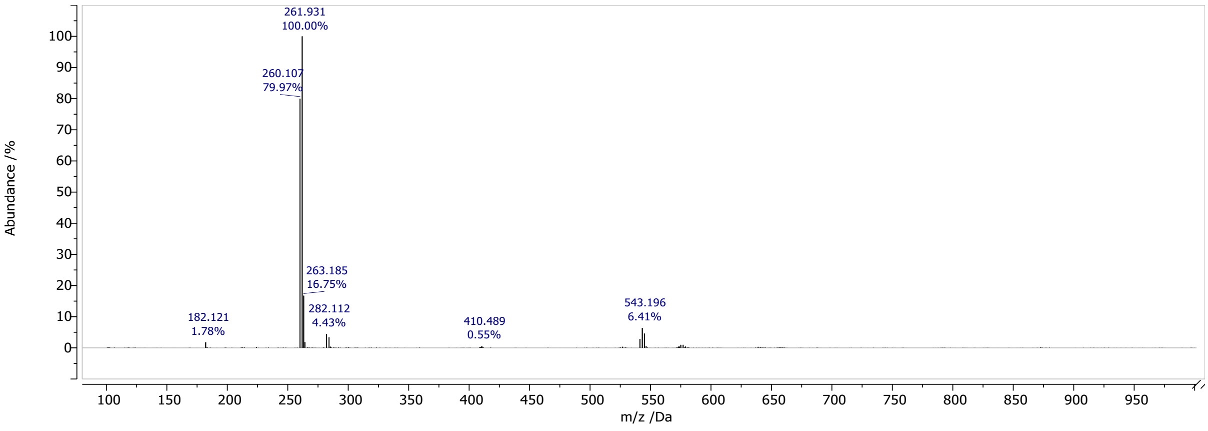

Figure S18: ES+ MS data for ethyl 4-bromo-6-(hydroxymethyl)picolinate.

2-(2,2-dibromovinyl)-1,3,5-trimethoxybenzene

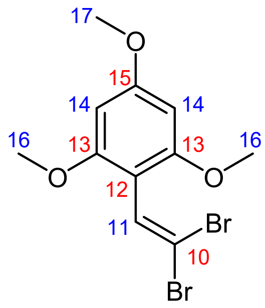

A solution of CBr_4 (1.69 g, 5.10 mmol) in anhydrous dichloromethane (3 cm^3) was dropwise added to a cooled (ice bath) solution of 2,4,6-trimethoxybenzaldehyde **7** (0.5 g, 2.5 mmol) and PPh_3 (5.36 g, 20.5 mmol) in anhydrous dichloromethane (3 cm^3), and the resulting solution was stirred (rt, 20 h, argon). After that, the solution was washed with deionised water (10 cm^3 $\times$ 5) followed by extraction of combined aqueous layers with dichloromethane (10 cm^3 $\times$ 3). The combined organic layers were dried over Na_2SO_4, filtered and concentrated. The crude product was purified by column chromatography (silica, 30 to 50 % dichloromethane in hexane) to yield white solid (321 mg, 0.912 mmol, 36%);

^{1}H NMR (400 MHz, $CDCl_3$) δ 7.19 (1 H, s, H^{11}), 6.11 (2 H, s, H^{14}), 3.83 (3 H, s, H^{17}), 3.81 (6 H, s, H^{16});

LCMS (ESI$^+$) *m/z* 351 $[M+H]^+$.

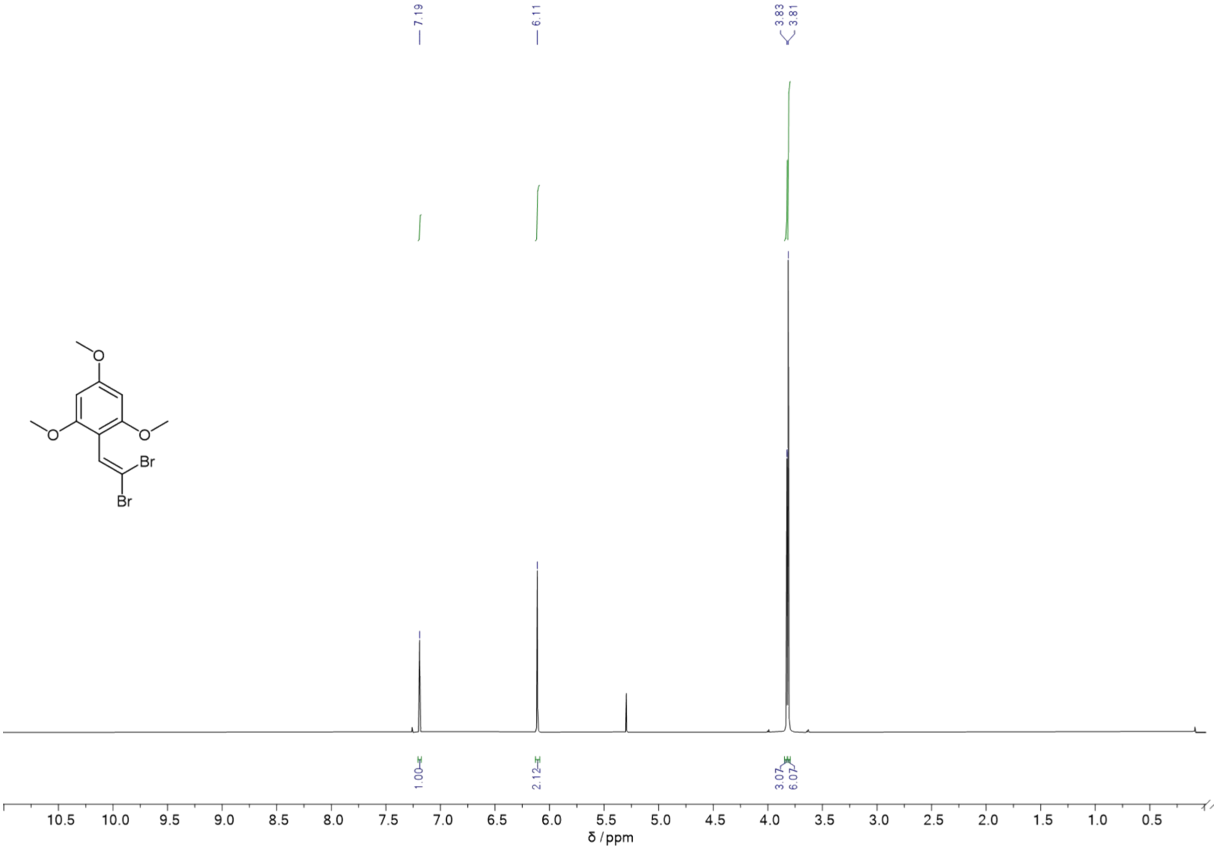

Figure S19: ^{1}H NMR (400 MHz, $CDCl_3$) spectrum of 2-(2,2-dibromovinyl)-1,3,5-trimethoxybenzene.

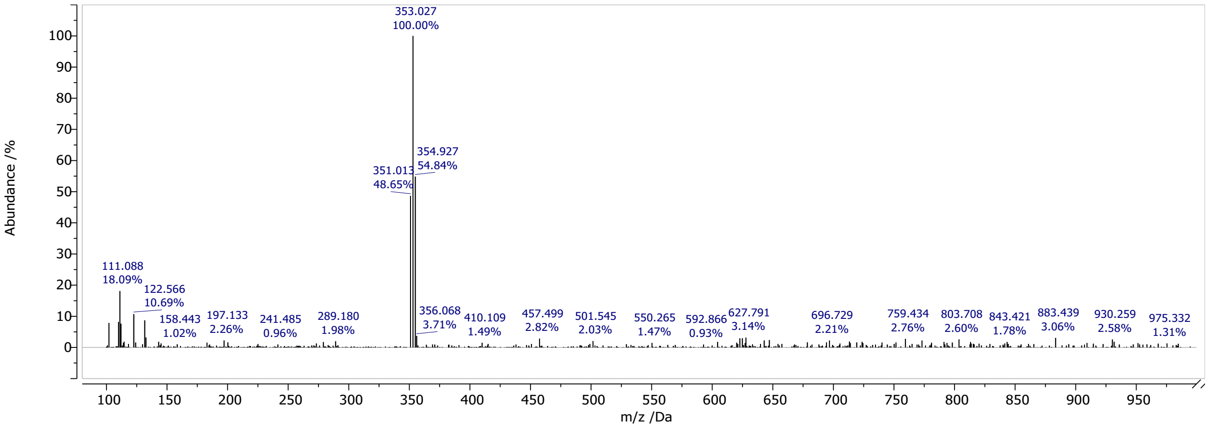

Figure S20: ES+ MS data for 2-(2,2-dibromovinyl)-1,3,5-trimethoxybenzene.

2-ethynyl-1,3,5-trimethoxybenzene

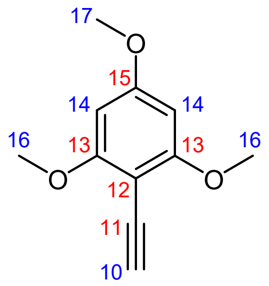

2-(2,2-dibromovinyl)-1,3,5-trimethoxybenzene **8** (321 mg, 0.912 mmol) was dissolved in anhydrous tetrahydrofuran and cooled to -78 °C (acetone, dry ice). 1.6 M solution of n-butyllithium in hexane (1.5 cm^3, 2.4 mmol) was then added dropwise, and the resulting orange solution was stirred (20 min). Deionised water (4 cm^3) was then added dropwise, and the resulting green solution was stirred (rt, 30 min). After that, the organic solvents were removed under reduced pressure, and the resulting aqueous solution was extracted with ethyl acetate (20 $cm^3 \times 3$). The combined organic layers were washed with brine, dried over Na_2SO_4, filtered and dried in vacuo to yield green solid that was used without further purification (0.171 g, 0.890 mmol, 98 %);

^{1}H NMR (400 MHz, $CDCl_3$) δ 6.10 (2 H, s, H^{14}), 3.88 (6 H, s, H^{16}), 3.83 (3 H, s, H^{17}), 3.50 (1 H, s, H^{10}).

LCMS (ESI$^+$) *m/z* 192 [M+H]$^+$.

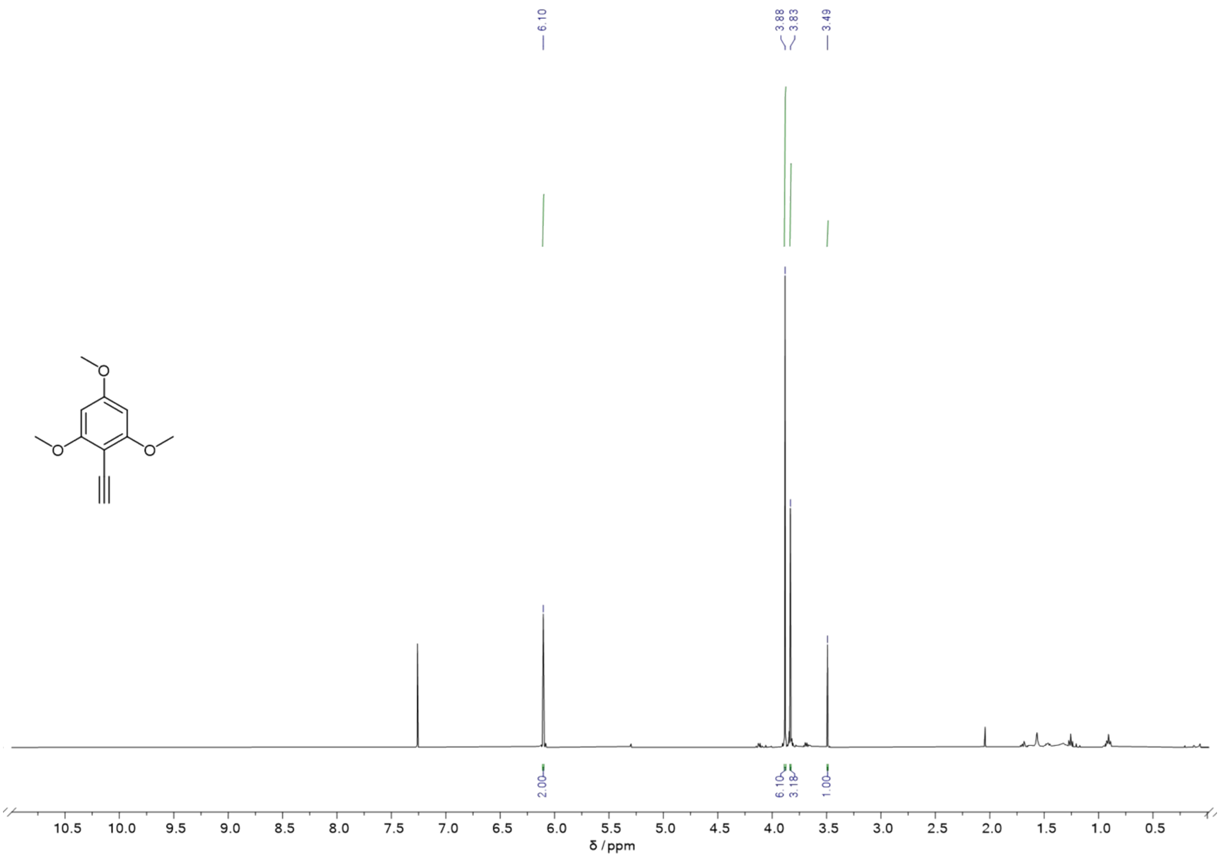

Figure S21: ^{1}H NMR (400 MHz, $CDCl_3$) spectrum of 2-ethynyl-1,3,5-trimethoxybenzene.

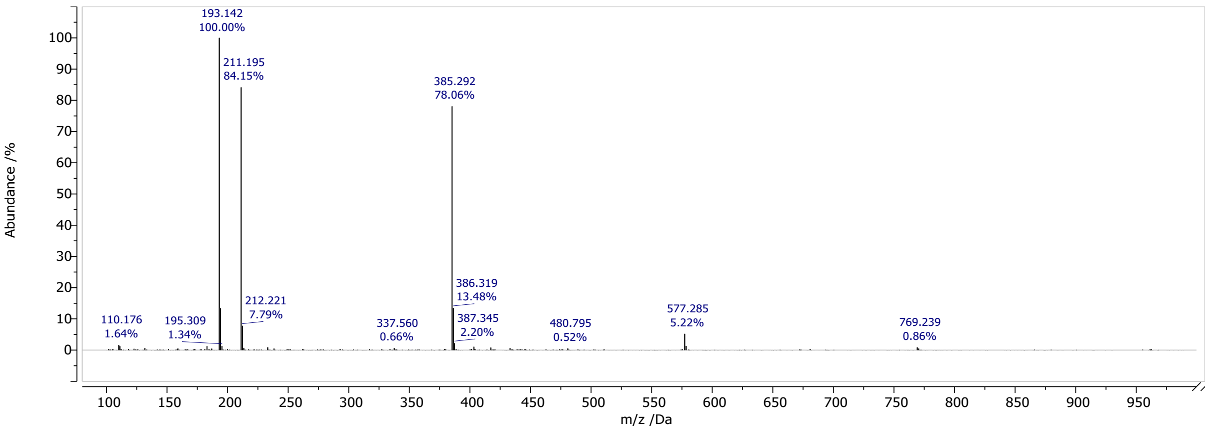

Figure S22: ES+ MS data for 2-ethynyl-1,3,5-trimethoxybenzene.

ethyl 6-(hydroxymethyl)-4-((2,4,6-trimethoxyphenyl)ethynyl)picolinate

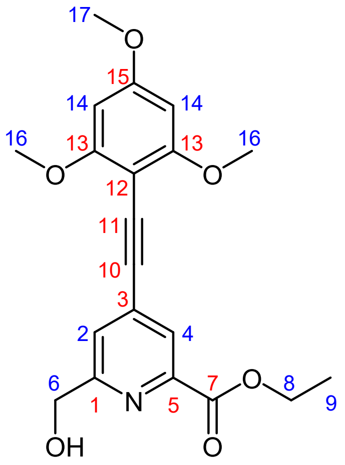

2-ethynyl-1,3,5-trimethoxybenzene (300 mg, 1.56 mmol, 1 eq.), diethyl 4-bromopyridine-2,6-dicarboxylate (446 mg, 1.72 mmol, 1.1 eq.) and $[Pd(allyl)Cl]_2$ (60 mg, 0.164 mmol, ~10 mol%) were dissolved in anhydrous acetonitrile (2 ml) and degassed. After that, triethylamine (2.16 ml, 15.6 mmol, 10 eq.) and piperidine (0.6 ml, 6.24 mmol, 4 eq.) were added, and the resulting solution was stirred (40°C, 12 h, argon). Organic solvents were then removed under reduced pressure, and the residue was dissolved in dichloromethane (10 ml). The resulting solution was washed with deionised water (5 ml × 3), and the combined aqueous phases were extracted with dichloromethane (5 ml × 3). The combined organic layers were dried over magnesium sulphate, filtered and isolated from the solvent under reduced pressure. The resulting yellow oil was purified by column chromatography (silica, 4% methanol in dichloromethane) to yield yellow solid (204 mg, 0.550 mmol, 35%).

^{1}H NMR (700 MHz, $CDCl_3$) δ 8.10 (1 H, d, $^4J_{H-H}$ 1.4, H^4), 7.58 (1 H, d, $^4J_{H-H}$ 1.4, H^2), 6.11 (2 H, s, H^{14}), 4.82 (2 H, s, H^6), 4.47-4.43 (2H, m, H^8), 3.90 (6 H, s, H^{16}), 3.85 (3 H, s, H^{17}), 1.42 (3 H, t, $^3J_{H-H}$ 7.1, H^9);

^{13}C NMR (700 MHz, $CDCl_3$) δ 164.86 (C^7), 162.87 (C^{15}), 162.82 (C^{13}), 159.92 (C^1), 147.14 (C^5), 134.48 (C^3), 125.61(C^4), 125.14 (C^2), 93.35 (C^{10}), 93.07 (C^{11}), 90.44 (C^{14}), 89.26 (C^{12}), 64.38 (C^6), 61.94 (C^8), 56.07 (C^{16}), 55.48 (C^{17}), 14.27 (C^9);

HRMS (ESI$^+$) *m/z* 372.1443 ($C_{20}H_{22}NO_6$ requires 372.1447).

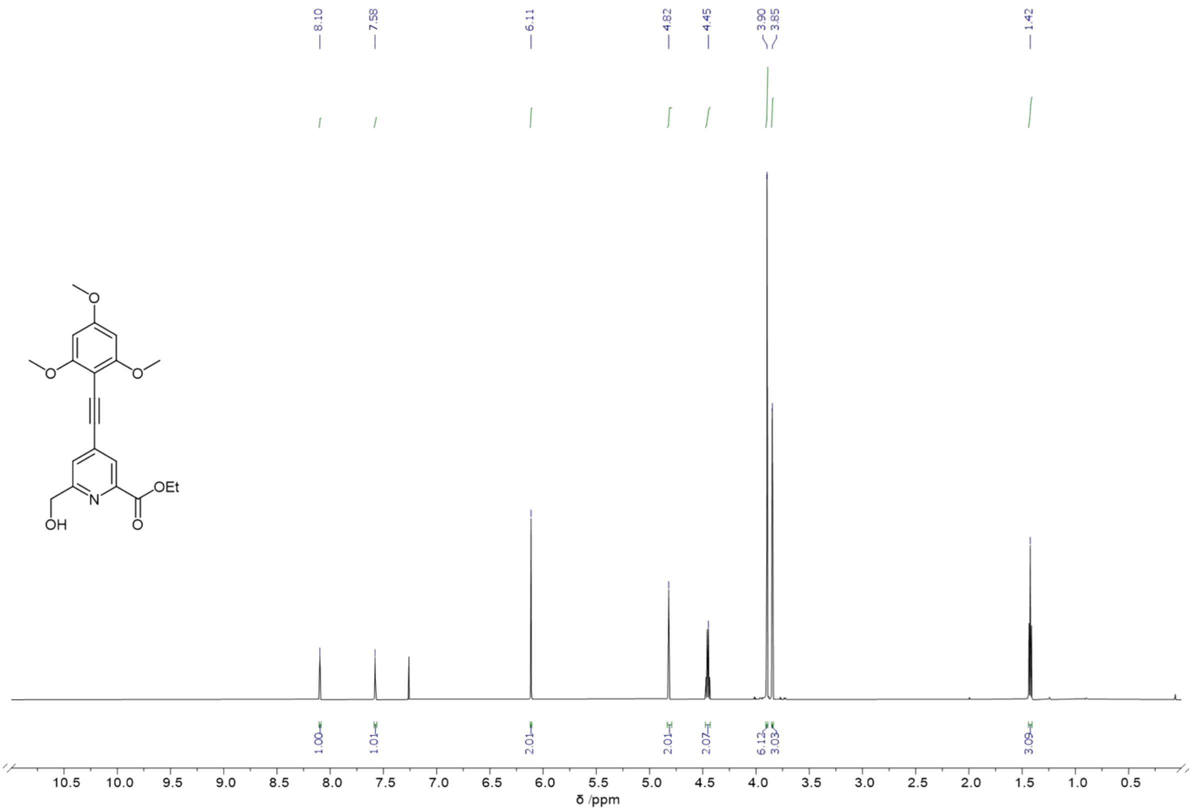

Figure S23: ^{1}H NMR (600 MHz, $CDCl_3$) spectrum of ethyl 6-(hydroxymethyl)-4-((2,4,6-trimethoxyphenyl)ethynyl)picolinate.

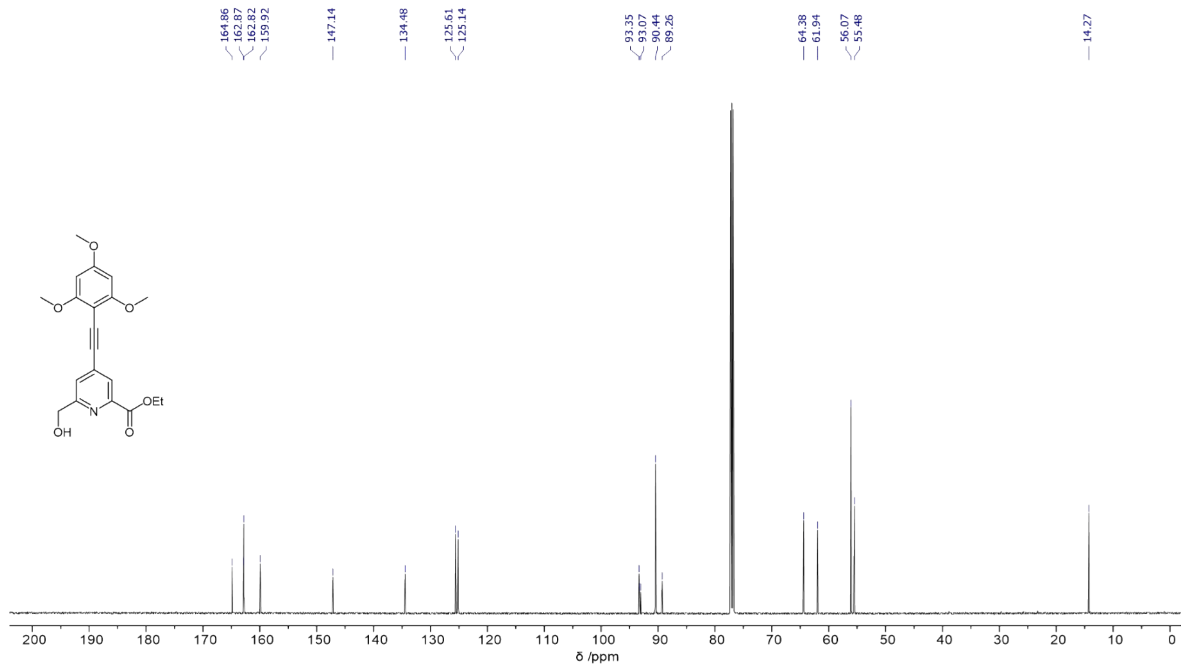

Figure S24: ^{13}C NMR (151 MHz, $CDCl_3$) spectrum of ethyl 6-(hydroxymethyl)-4-((2,4,6-trimethoxyphenyl)ethynyl)picolinate.

Single Mass Analysis

Tolerance = 3.0 mDa / DBE: min = -1.5, max = 50.0
Element prediction: Off
Number of isotope peaks used for i-FIT = 5

Monoisotopic Mass, Even Electron Ions
1158 formula(e) evaluated with 5 results within limits (up to 200 closest results for each mass)
Elements Used:
C: 0-40 H: 0-60 N: 0-6 O: 0-10 P: 0-3

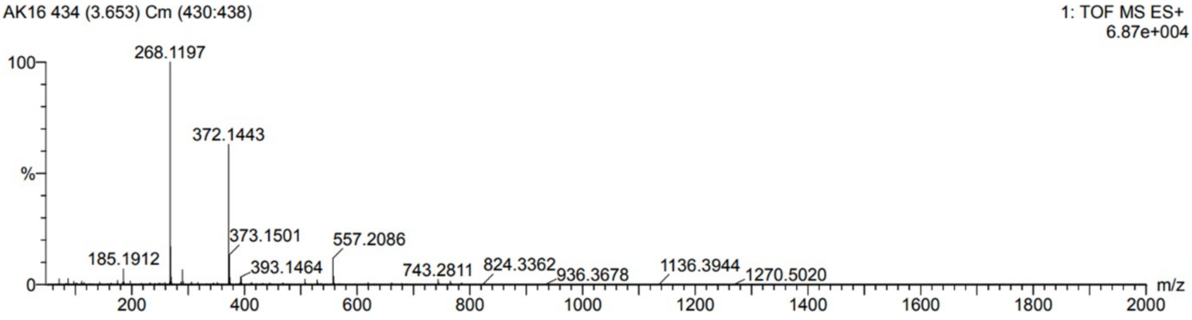

Minimum:				-1.5			
Maximum:		3.0	5.0	50.0			
Mass	Calc. Mass	mDa	PPM	DBE	i-FIT	i-FIT (Norm)	Formula
372.1443	372.1447	-0.4	-1.1	10.5	755.9	0.1	C20 H22 N O6

Figure S25: HRMS data for ethyl 6-(hydroxymethyl)-4-((2,4,6-trimethoxyphenyl)ethynyl)picolinate.

ethyl 6-(((methylsulfonyl)oxy)methyl)-4-((2,4,6-trimethoxyphenyl)ethynyl)picolinate

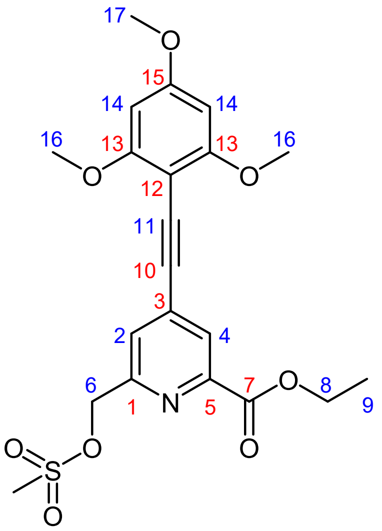

Ethyl 6-(hydroxymethyl)-4-((2,4,6-trimethoxyphenyl)ethynyl)picolinate (119 mg, 0.320 mmol, 1eq.) was dissolved in anhydrous tetrahydrofuran (3.5 ml). *N*,*N*-Diisopropylethylamine (0.17 ml, 0.960, 3 eq.) and methanesulfonic anhydride (0.223 g, 1.280 mmol, 4 eq.) were added, and the resulting solution was stirred (rt, 4h, argon) and monitored by TLC (silica, 5% methanol in dichloromethane). Organic solvents were then evaporated under reduced pressure, and the residue dissolved in dichloromethane (10 ml). The resulting solution was washed with deionised water (10 ml × 3), and the combined aqueous phases were extracted with dichloromethane (5 ml × 3). The combined organic layers were dried over magnesium sulphate, filtered and isolated from the solvent under reduced pressure to yield pale yellow solid that was used in the next step without further purification.

^{1}H NMR (400 MHz, $CDCl_3$) ^{1}H NMR (400 MHz, $CDCl_3$) δ 8.17 (1 H, s, H^4), 7.72 (1 H, s, H^2), 6.12 (2 H, s, H^{14}), 5.44 (2 H, s, H^6), 4.58-4.47 (2H, m, H^8), 3.91 (6 H, s, H^{16}), 3.86 (3 H, s, H^{17}), 1.44 (3 H, t, $^{3}J_{H\text{-}H}$ 7.5, H^9).

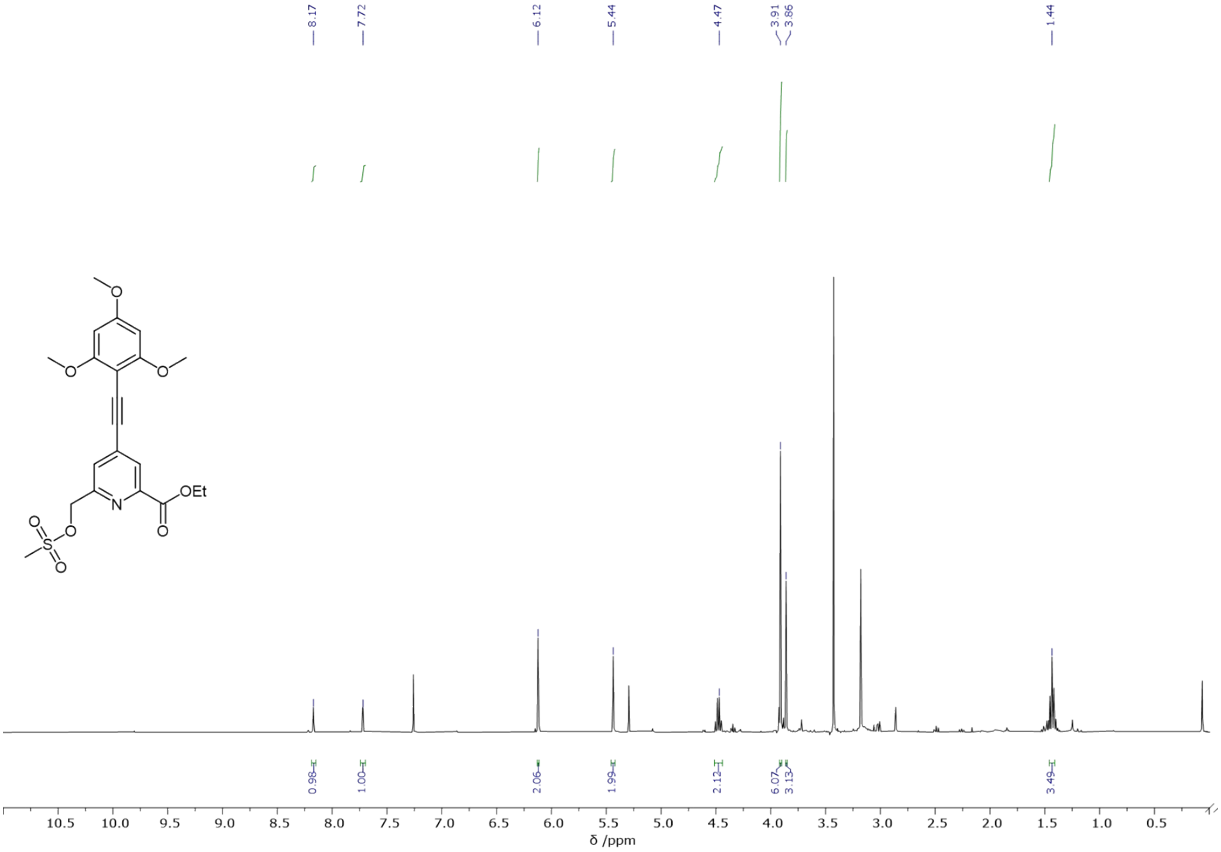

Figure S26: ^{1}H NMR (400 MHz, $CDCl_3$) spectrum of ethyl 6-(((methylsulfonyl)oxy)methyl)-4-((2,4,6-trimethoxyphenyl)ethynyl)picolinate.

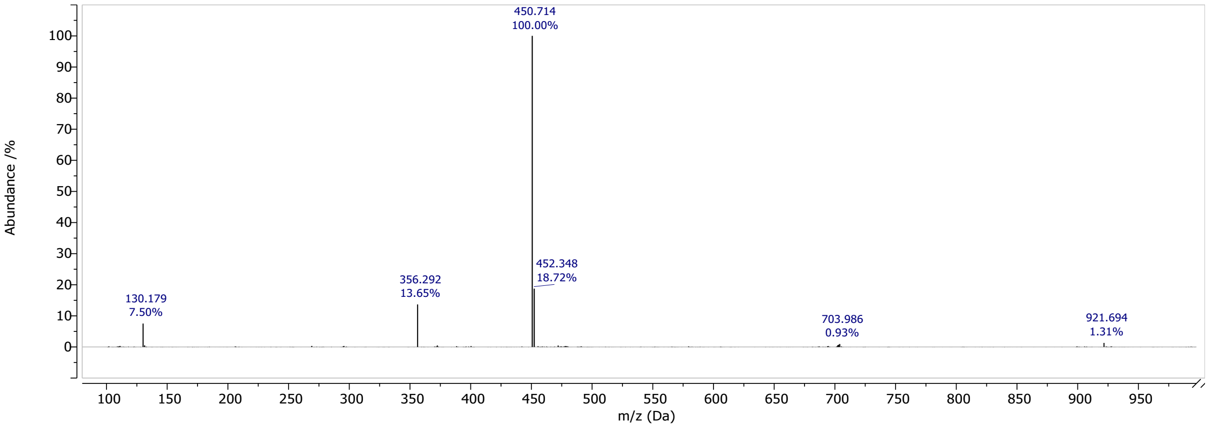

Figure S27: HRMS data for ethyl 6-(((methylsulfonyl)oxy)methyl)-4-((2,4,6-trimethoxyphenyl)ethynyl)picolinate.

triethyl 6,6',6''-((1,4,7-triazonane-1,4,7-triyl)tris(methylene))tris(4-((2,4,6-trimethoxyphenyl)ethynyl)picolinate)

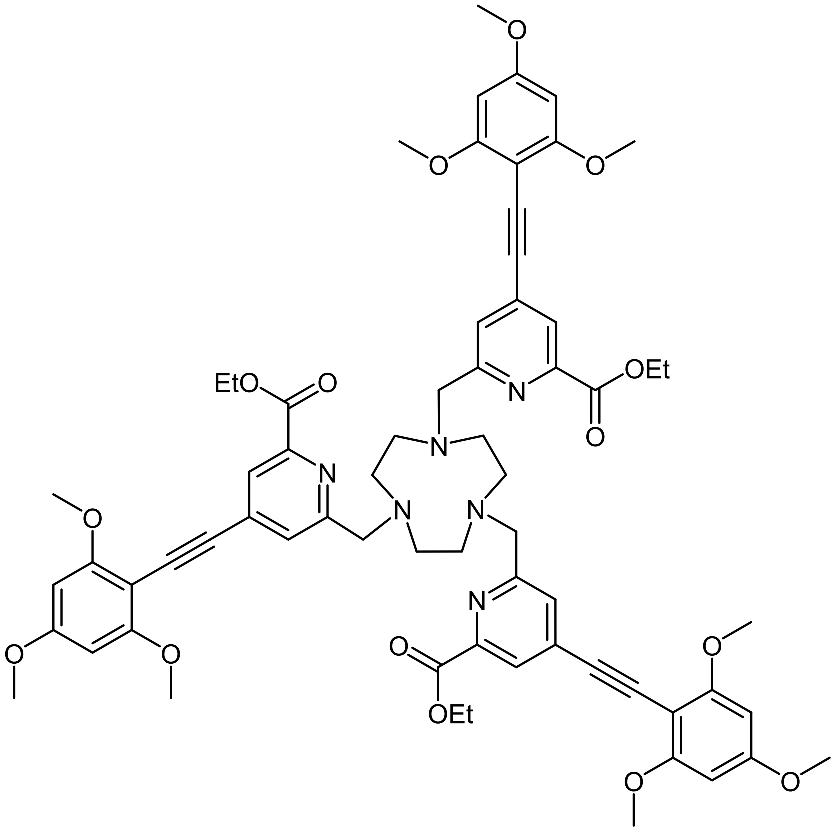

Ethyl 6-(((methylsulfonyl)oxy)methyl)-4-((2,4,6-trimethoxyphenyl)ethynyl)picolinate (*0.144 g, 0.320 mmol,* 3.5 eq.), TACN•3HCl (22 mg, 0.0914 mmol, 1 eq.) and potassium carbonate (88 mg, 0.6398 mmol, 7 eq.) were dissolved in anhydrous acetonitrile (5 ml) and stirred (60°C, 12 h, argon). The crude was isolated from the solvent under reduced pressure and dissolved in dichloromethane. It was then washed with deionised water (5 ml × 3), and the combined aqueous phases were extracted with dichloromethane (5 ml × 3). The combined organic layers were dried over magnesium sulphate, filtered and isolated from the solvent under reduced pressure to yield pale yield yellow solid (*0.0914 mmol*) that was used in the next step without further purification.

HRMS (ESI$^+$) *m/z* 1189.5195 $[M+H]^+$ ($C_{66}H_{72}N_6O_{15}$ requires 1189.5134).

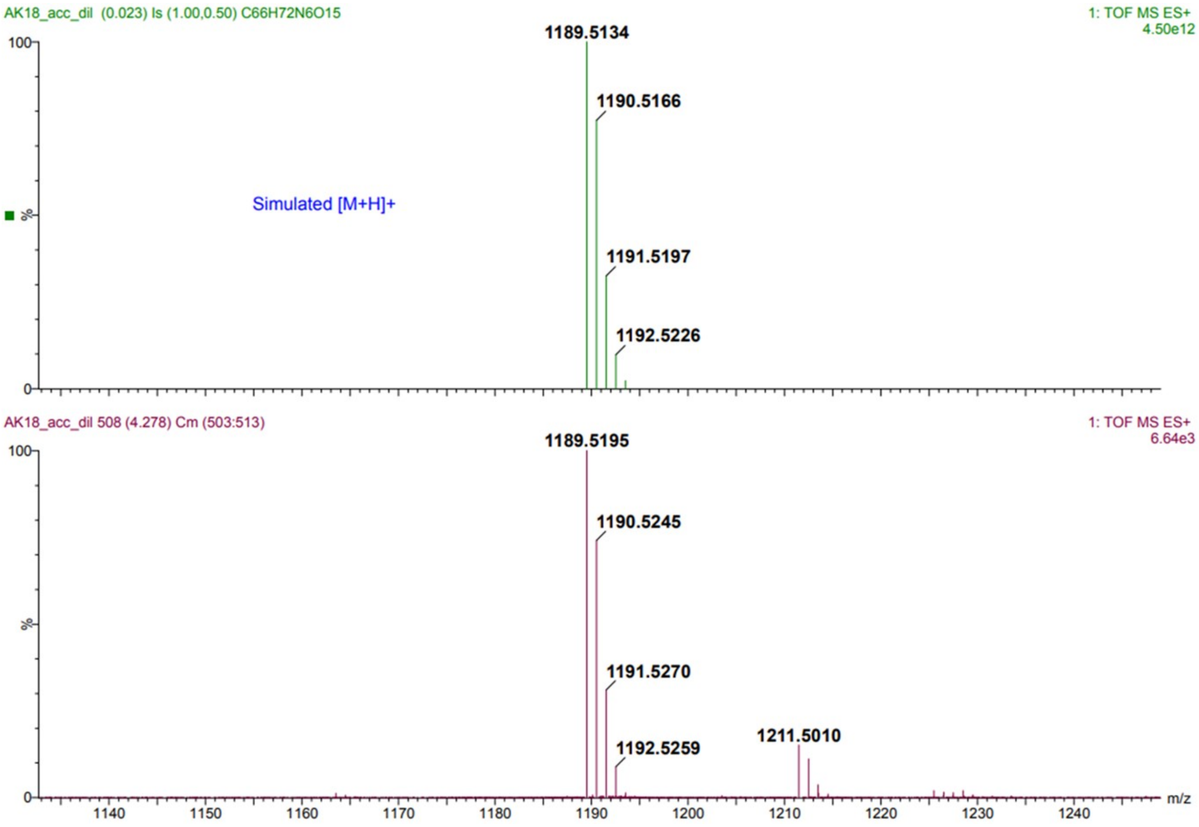

Figure S28: HRMS data for triethyl 6,6',6''-((1,4,7-triazonane-1,4,7-triyl)tris(methylene))tris(4-((2,4,6-trimethoxyphenyl)ethynyl)picolinate).

EuL

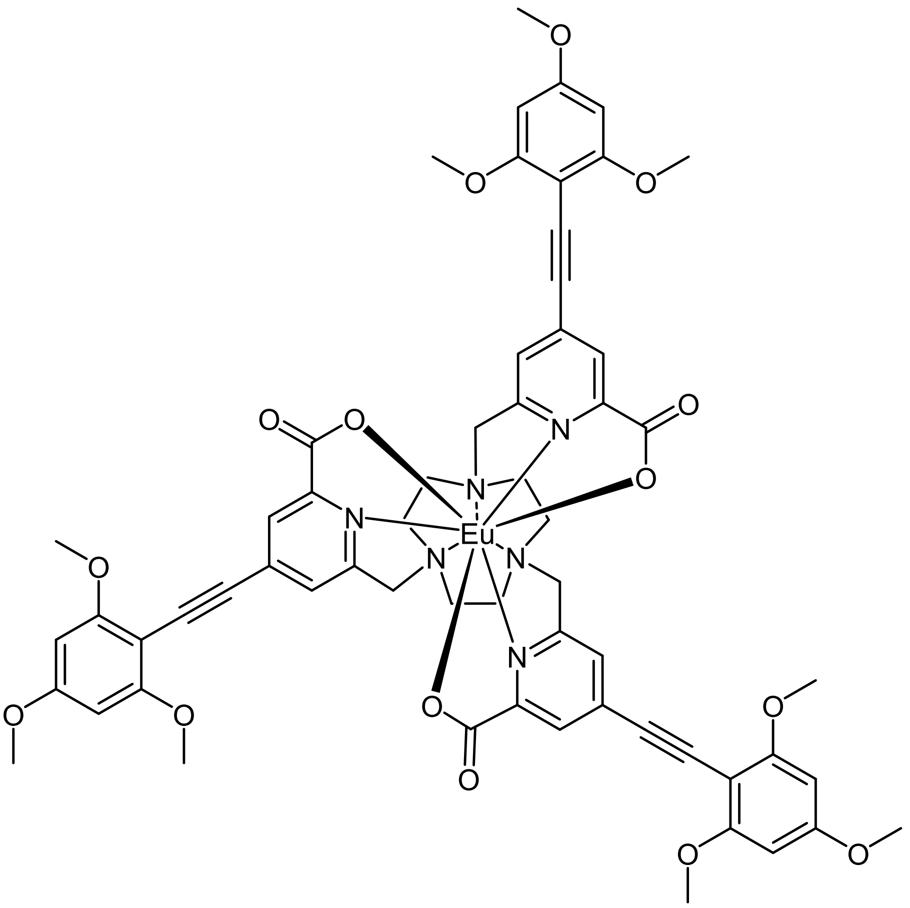

Triethyl 6,6',6''-((1,4,7-triazonane-1,4,7-triyl)tris(methylene))tris(4-((2,4,6-trimethoxy phenyl)ethynyl)picolinate) (*0.0914 mmol*) was dissolved in methanol (5 ml) and deionised water until white precipitate remained undissolved. pH of the resulting solution was adjusted to 12.5 using solution of sodium hydroxide in methanol. The solution was stirred (rt, 72 h), while its pH was monitored and periodically adjusted to 12.5 until hydrolysis completion was confirmed by LCMS. After that, the solution pH was readjusted to 6.5 using 1 M solution of hydrochloric acid. $EuCl_3 \bullet 6H_2O$ was then added (37 mg, 0.101 mmol, 1.1 eq.) and the resulting solution was stirred (rt, 18 h). Solvent was then removed under reduced pressure to yield pale yellow solid which was redissolved in dichloromethane (10 ml). The resulting solution was washed with deionised water (5 ml × 3), and the combined aqueous phases were extracted with dichloromethane (5 ml × 3). The combined organic layers were dried over magnesium sulphate, filtered and isolated from the solvent under reduced pressure to yield pale yield yellow solid that was purified using was purified using reverse-phase HPLC (10 to 100% acetonitrile in water, retention time = 11.4 mins) to yield yellow solid (67 mg, 58% over 3 steps); HRMS (ESI+) *m/z* 1253.3190 $[M+H]^+$ ($C_{60}H_{57}EuN_6O_{15}$ requires 1253.3159).

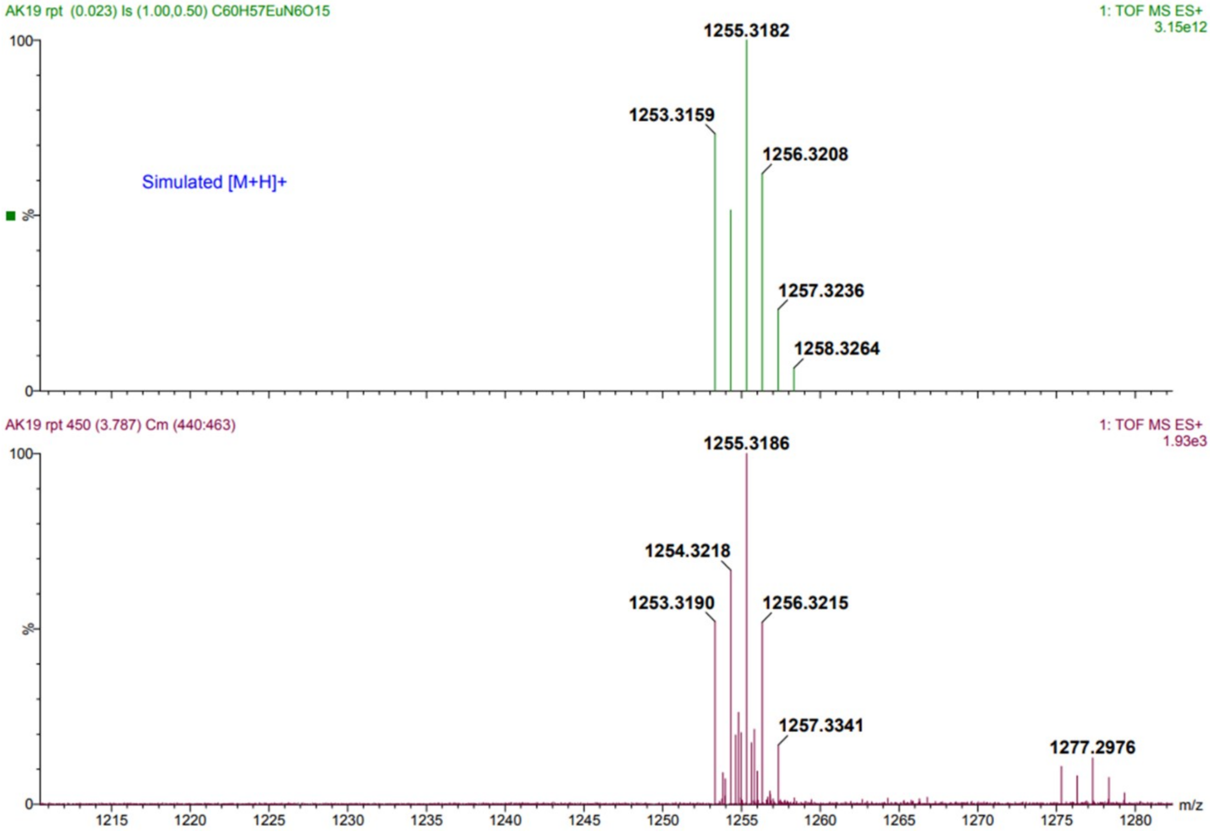

Figure S29: HRMS data for EuL.

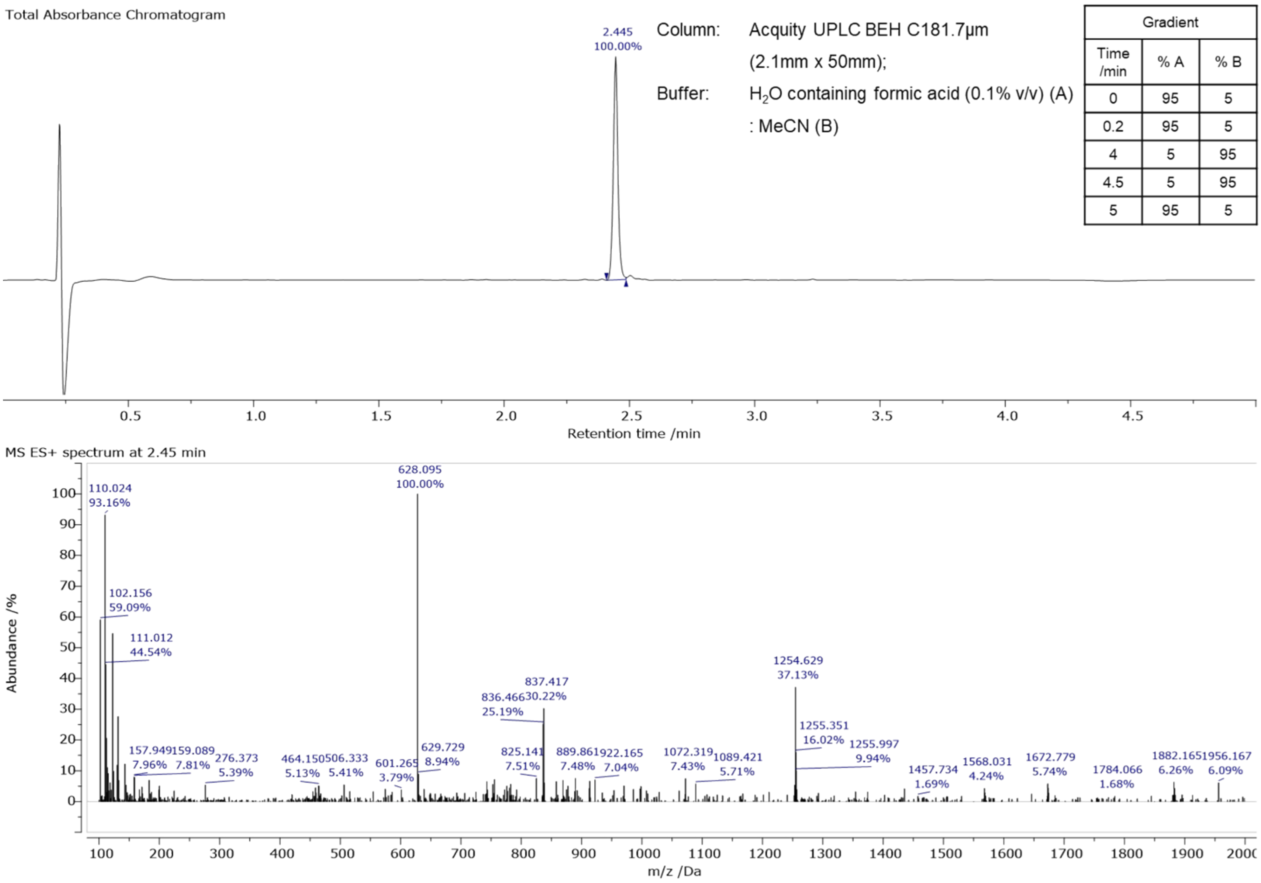

Gradient		
Time /min	% A	% B
0	95	5
0.2	95	5
4	5	95
4.5	5	95
5	95	5

Figure S30: Total absorbance chromatogram (top) and the ES+ MS spectrum at 2.445 min (bottom) for the sample of EuL.

Supplementary References

1 R. Carr, R. Puckrin, B. K. McMahon, R. Pal, D. Parker and L.-O. Pålsson, Induced circularly polarized luminescence arising from anion or protein binding to racemic emissive lanthanide complexes, *Methods Appl. Fluoresc.*, 2014, **2**, 024007.

2 J. D. Fradgley, A. T. Frawley, R. Pal and D. Parker, Striking solvent dependence of total emission and circularly polarised luminescence in coordinatively saturated chiral europium complexes: solvation significantly perturbs the ligand field, *Physical Chemistry Chemical Physics*, 2021, **23**, 11479–11487.

3 C. Xu and W. W. Webb, Measurement of two-photon excitation cross sections of molecular fluorophores with data from 690 to 1050 nm, *J. Opt. Soc. Am. B*, 1996, **13**, 481.

4 L.-O. Pålsson, R. Pal, B. S. Murray, D. Parker and A. Beeby, Two-photon absorption and photoluminescence of europium based emissive probes for bioactive systems, *Dalton Trans.*, 2007, 5726.

5 P. Stachelek, L. MacKenzie, D. Parker and R. Pal, Circularly polarised luminescence laser scanning confocal microscopy to study live cell chiral molecular interactions, *Nat Commun*, 2022, **13**, 553.
